# mTh1 driven expression of hTDP-43 results in typical ALS/FTLD neuropathological symptoms

**DOI:** 10.1371/journal.pone.0197674

**Published:** 2018-05-22

**Authors:** Barbara Scherz, Roland Rabl, Stefanie Flunkert, Siegfried Rohler, Joerg Neddens, Nicole Taub, Magdalena Temmel, Ute Panzenboeck, Vera Niederkofler, Robert Zimmermann, Birgit Hutter-Paier

**Affiliations:** 1 QPS Austria GmbH, Grambach, Austria; 2 Karl-Franzens University, Institute of Molecular Biosciences, Graz, Austria; 3 Medical University Graz, Institute of Pathophysiology and Immunology, Graz, Austria; International Centre for Genetic Engineering and Biotechnology, ITALY

## Abstract

Transgenic mouse models are indispensable tools to mimic human diseases and analyze the effectiveness of related new drugs. For a long time amyotrophic lateral sclerosis (ALS) research depended on only a few mouse models that exhibit a very strong and early phenotype, e.g. SOD1 mice, resulting in a short treatment time window. By now, several models are available that need to be characterized to highlight characteristics of each model. Here we further characterized the mThy1-hTDP-43 transgenic mouse model TAR6/6 that overexpresses wild type human TARDBP, also called TDP-43, under control of the neuronal Thy-1 promoter presented by Wils and colleagues, 2010, by using biochemical, histological and behavioral readouts. Our results show that TAR6/6 mice exhibit a strong TDP-43 expression in the hippocampus, spinal cord, hypothalamus and medulla oblongata. Apart from prominent protein expression in the nucleus, TDP-43 protein was found at lower levels in the cytosol of transgenic mice. Additionally, we detected insoluble TDP-43 in the cortex, motoneuron loss, and increased neuroinflammation in the central nervous system of TAR6/6 animals. Behavioral analyses revealed early motor deficits in the clasping- and wire suspension test as well as decreased anxiety in the elevated plus maze. Further motor tests showed differences at later time points compared to non-transgenic littermates, thus allowing the observation of onset and severity of such deficits. Together, TAR6/6 mice are a valuable tool to test new ALS/FTLD drugs that target TDP-43 expression and insolubility, neuroinflammation, motoneuron loss or other TDP-43 related downstream signaling pathways since these mice exhibit a later pathology as previously used ALS/FTLD mouse models.

## Introduction

The transactive response DNA-binding protein 43 (TARDBP; TDP-43) has been identified to play a crucial role in the development of neurodegenerative diseases, especially in amyotrophic lateral sclerosis (ALS) and frontotemporal lobar degeneration with ubiquitin-positive inclusions (FTLD-U). TDP-43 protein has been recognized to be the main component of ubiquitinated inclusions in human ALS and FTLD samples [1,2] and FTLD-U was since renamed FTLD-TDP.

Physiological TDP-43 is ubiquitously expressed and mainly localized in the nucleus. The protein is synthesized in the cytoplasm but shuttles between the nucleus and cytoplasm [3–5]. TDP-43 can bind DNA as well as RNA [6,7] and seems to be critical for fat metabolism and embryonic stem cell survival [8]. Pathological TDP-43 is highly ubiquitinated [1], hyperphosphorylated [9], and mislocated to the cytoplasm [1]. Truncated isoforms of TDP-43 have been found in the telencephalon of ALS patients suggesting that TDP-43 is prone to be cleaved. In addition, disease-related TDP-43 is relatively insoluble, a feature that might be caused by hyperphosphorylation [1]. Insolubility of TDP-43 might furthermore support aggregation of the protein in the cytoplasm, suggesting not only a loss of function but also a gain of toxic function (discussed in [10]). Currently, 47 missense and one truncating *TARDBP* gene mutations have been reported (for review see [11]), most of them located in the glycine-rich domain causing predominantly ALS and only rarely FTLD (summarized in [10]). The distribution pattern of TDP-43 inclusions can be distinguished between ALS and FTLD-TDP patients. ALS patients exhibit strong TDP-43 pathology in the motor cortex, spinal cord and basal ganglia, while FTLD-TDP patients show TDP-43 expression throughout the central nervous system (CNS), except for the occipital cortex and cerebellum [12]. In ALS, TDP-43 histopathology is characterized by cytoplasmic inclusions of skein-like or compact granular shape and at the same time a complete absence of TDP-43 in the nucleus [1,12].

Since 2009, a whole series of genetically altered TDP-43 mouse models were generated (for review see [13,14]). These models express wild type or mutated TDP-43 under the control of different promoters, such as the prion or Thy-1 promoter, constitutionally or conditionally [15–20]. A major drawback of most of these models is that some barely show any phenotype [16,18] and others have such a strong and early phenotype resulting in severely shortened survival times [15–18], so that neither type of these models is suitable for treatment studies designed to characterize new compounds against ALS/FTLD. In 2010, Wils and colleagues generated two transgenic ALS mouse models, homozygous TAR4/4 and TAR6/6. Both founder lines express wild type human TDP-43 (hTDP-43) under control of the neuron specific Thy-1.2 promoter [17]. While the TAR4/4 model presents a very strong and early phenotype with an average survival time of 25 days, the TAR6/6 model develops a later phenotype onset and an average survival time of 6.5 months [17]. TAR6/6 mice were shown to have 1.2 fold increased hTDP-43 expression levels compared to murine TDP-43, an abnormal hind limb reflex at two months, and disturbed motor behavior at approximately four months of age. Additionally, histological analyses revealed neuronal loss and neuroinflammation in the motor cortex as well as in the spinal cord of 6 months old TAR6/6 mice. These results already indicate that TAR6/6 mice exhibit an ALS/FTLD like phenotype with a moderate progression rate. To be able to use TAR6/6 mice for ALS/FTLD compound tests, we validated TDP-43 expression in the spinal cord and further characterized this model for histopathological and biochemical features and motor deficits for the first time at presymptomatic, symptomatic and late disease stages. Our results verify and extend the results by Wils and colleagues and emphasize the strength of TAR6/6 mice as a valid ALS/FTLD model and its suitability to test new compounds against these devastating, incurable diseases.

## Materials & methods

### Animals

TAR6 mice and corresponding littermates were provided by ‘Neurodegenerative Brain Diseases Group’, Department of Molecular Genetics, VIB, Antwerp, Belgium [17]. TAR6/6 transgenic mice express human wild type TDP-43 under the control of the murine Thy-1.2 promoter, mThy1-hTDP-43 mice [17]. Homozygous mice (TAR6/6) were bred by mating hemizygous mice (TAR6). Non-transgenic (ntg) littermates served as control for all experiments. Equal numbers of male and female TAR6/6, TAR6 and ntg mice were used and kept in individually ventilated cages under a 12 hour light/dark cycle at the QPS Austria, AAALAC accredited and pathogen free animal facility. Animals were provided with standard rodent chow (Altromin^™^, Germany) normal tap water *ad libitum* and a 50 mm square piece of pressed cotton (nestlets) as nesting material. Room temperature and humidity were kept at approximately 24°C and 40–70%, respectively. Before starting the experiments animals were group-housed, but since mice had to be single-housed for the first behavioral test, all animals were kept single housed from that time on to prevent fighting between male groups caused by regrouping. Single housing is shown to influence several aspects of the mouse phenotype like body weight, activity and anxiety but also striatal changes in dopamine and serotonin turnover levels [21], therefore male and female mice were single housed after the nest building test to keep housing conditions equal between sexes. Since all animals were housed equally, possible alterations should be similar between genotypes. Mice of four cohorts were analyzed: The first cohort was analyzed for behavioral deficits at the age of 1.5 months and afterwards analyzed by biochemical and histological methods. The second cohort was analyzed longitudinally from the age of 3 to 4.75 months. The third and fourth cohorts were only analyzed by biochemical and histological methods at the age of 3 and 6 months, respectively. Actual animal numbers are given in the figure legends. Animal studies conformed to the Austrian guidelines for the care and use of laboratory animals and were approved by the Styrian government, Austria (FA10A-78Jo-69-2010, ABT13-78Jo168-2015). All efforts were made to minimize suffering.

### Behavioral tests

All behavioral tests were performed during the early phase of the light cycle. Animals were allocated to the corresponding experimental group according to their genotype. The following behavioral tests were performed in week 6 (cohort I) or repeatedly from week 12, 14–17 (cohort II) in the following order: nest building, clasping, wire suspension, Rota Rod. Additionally, animals were tested in the elevated plus maze and fear conditioning test in week 18 (cohort II). Hemizygous mice were analyzed at the age of 1.5, 9, or 12 months.

The nest building test was performed as previously described [22].

To analyze animals for the hind limb reflex, mice were lifted on the base of their tail and clasping was scored. Healthy mice spread their hind limbs but with increasing pathology mice press their hind limbs against each other. The hind limb position was scored as follows: Score 3: mice with normal hind limb position. Score 2: mice with intermediate hind limb position. Score 1: mice with closed hind limb position [17].

The wire suspension test and Rota Rod test were conducted as previously described [23].

The elevated plus maze test analyzes the anxiety of mice. The animal was placed in the center of a four-arm-maze from which it can choose any of the four narrow arms. Two arms are well lit and open. The two alternating arms are enclosed with walls and dimmed. Healthy rodents will prefer the closed arms but will venture out into the open arms. The time, animals spent in the different arms was measured by automated video-tracking software (Videomot, TSE-Systems, Germany).

### Tissue sampling

All mice were sedated by standard inhalation anesthesia (isofluorane). Once animals were deeply anesthetized, mice were transcardially perfused with physiological (0.9%) saline. Thereafter, spinal cords, including vertebrae, and brains were rapidly removed and brains were hemisected. The spinal cords of half of the animals and the right hemispheres of all animals were fixed by immersion at 4°C in freshly prepared 4% formaldehyde in phosphate buffer (pH 7.4) for 2 or 24 hours, respectively. After cryo-conservation in a 15% sucrose/phosphate buffered saline (PBS) solution, right hemispheres were shock frozen in dry-ice cooled liquid isopentane. Spinal cords and the right hemispheres were than stored at -80°C until used for histological analyses. The spinal cords were further dissected from vertebrae, embedded in paraffin and stored at room temperature.

The spinal cords of half of the animals and the left hemispheres of all animals that were used as whole or dissected into cortex, hippocampus and midbrain, were shock frozen on dry ice and stored at -80°C until used for biochemical analyses.

### Homogenization of frozen tissue samples

The frozen, unfixed tissue samples [brain (left hemisphere), hippocampus and spinal cord] were weighed and homogenized for 20 seconds using the highest level of the Tissue Ruptor (Qiagen, Germany) in 5 volumes per weight RIPA buffer (50 mM Tris-HCl, pH 7.4, 1 mM EDTA, 150 mM NaCl, 1% NP-40 or 0.1% Triton-X, 2% SDS, 1 μM NaF, 0.2 mM sodium deoxycholate, 80 μM glycerophosphate 1x protease inhibitor (Calbiochem, Germany), 1x phosphatase inhibitor (Sigma, USA).

Samples were further analyzed by Western blotting using antibodies against human-TDP-43 (1:1,000, Abnova, Germany, H00023435-M01), total TDP-43 (1:5,000, Proteintech, USA 10782-2-AP), or β-tubulin isotype III (1:5,000, Sigma-Aldrich, USA, T8660). Protein levels of tTDP-43, hTDP-43 and ß-tubulin were densitometrically quantified using Image J. For densitometric quantification, protein signals of tTDP-43, hTDP-43 and truncated TDP-43 were analyzed by Image J.

### Cell fractionation

Two mid brain and 2 cortex samples of 4 ntg, 4 TAR6 and 4 TAR6/6 mice at an age of 3 months were fractionated into cytoplasm and nucleus using NE-PER^™^ Nuclear and Cytoplasmic Extraction Reagent (Thermo Scientific, USA #78833). Fractionation was performed according to the manual. Samples were additionally analyzed by Western blotting using antibodies against total TDP-43 (1:5,000, Proteintech, USA 10782-2-AP), human-TDP-43 (1:1,000, Abnova, Germany, H00023435-M01), glyceraldehyde-3-phosphate-dehydrogenase (GAPDH, 1:10,000, Sigma-Aldrich, USA, G9545) as marker for cytoplasmic fraction and HDAC3 (1:250, Abcam, UK, ab7050) as marker for nuclear fraction. Protein levels of tTDP-43, hTDP-43 and truncated TDP-43 were densitometrically quantified using Image J.

### TDP-43 fractionation

Five frozen brain samples of 3 months old ntg or TAR6/6 mice were thawed on ice and sonicated in 5x v/w RIPA buffer (50 mM Tris, 150 mM NaCl, 1% NP-40, 5 mM EDTA, 0.5% sodium deoxycholate, and 0.1% SDS, pH 8.0) containing protease and phosphatase inhibitor cocktails (Sigma, USA). Afterwards, samples were centrifuged at 100,000 g for 35 min at 4°C and supernatants collected as RIPA soluble fractions. The pellets were washed with RIPA buffer and sonicated in 2x (v/w) urea buffer (7 M urea, 2 M thiourea, 4% CHAPS, and 30 mM Tris, pH 8.5). After centrifugation at 100,000 g for 35 min at 22°C the supernatants were taken as RIPA insoluble (urea soluble) fractions. Samples were further analyzed by Western blotting using antibodies against total TDP-43 (1:5,000, Proteintech, USA 10782-2-AP), human-TDP-43 (1:1,000, Abnova, Germany, H00023435-M01), or GAPDH (1:10,000, Sigma-Aldrich, USA, G9545) as loading control. Protein levels of tTDP-43, hTDP-43 and truncated TDP-43 were densitometrically quantified using Image J.

### Histology

Frozen brain hemispheres and paraffin-embedded spinal cords were cut sagittally or transversely, respectively. Unless indicated otherwise, a systematic uniform random set of five mounted 10 μm thick brain sections from five medio-lateral levels and three mounted 5 μm thick spinal cord sections per animal were used for immunofluorescent labeling.

#### TDP-43 labeling

Sagittal sections were treated for 15 min with citrate buffer at 95°C and a 1 mg/ml sodium borohydride/PBS solution and subsequently blocked with 10% horse serum for one hour. Thereafter, sections were labeled with a human-TDP-43 antibody (1: 2,000; Proteintech, USA, 60019-2-Ig, monoclonal). Binding of the primary antibody was visualized using a highly cross-absorbed DyLight secondary antibody (DyLight650, 1:500, Jackson ImmunoResearch, USA, 712-495-153).

#### Co-labeling of human and total TDP-43

Sagittal sections were treated for 15 min with citrate buffer at 95°C and a 1 mg/ml sodium borohydride/PBS solution and subsequently blocked with M.O.M. Blocking Reagent in 0.3% Triton X-100/PBS for one hour at room temperature. Thereafter, sections were labeled with a human-TDP-43 antibody (1:1,500; Abnova, Taiwan, H00023435-M01, monoclonal), total TDP-43 antibody (1:400; Proteintech, USA, 10782-2-AP, polyclonal) and NeuN antibody (1:2,000, Synaptic Systems, Germany, 266 004, polyclonal) over night at room temperature. Binding of the primary antibody was visualized using highly cross-absorbed Alexa Fluor and DyLight secondary antibodies (Alexa Fluor555; 1:500; Abcam, USA, ab150110; DyLight650, 1:500, Abcam, USA, ab96922; Alexa Fluor 488, 1:500, Jackson ImmunoResearch, USA, 706-545-148) for 1 hour at room temperature followed by nuclear staining with 4’,6-Diamidin-2-phenylindol (DAPI, PanReac AppliChem GmbH, Germany, A1001) for 15 minutes.

#### GFAP and CD11b labeling

Sagittal sections were treated with a 1 mg/ml sodium borohydride/PBS solution followed by 1% hydrogen peroxide in methanol. To block unspecific labeling sections were treated with 10% donkey serum for 30 min. Further, sections were incubated with a GFAP (1:500; Dako, USA, Z0334) and CD11b antibody (1:1,000, BioRad Laboratories, USA, MCA711) for 1 h at room temperature. Binding of primary antibodies was visualized using highly cross-absorbed DyLight secondary antibodies (DyLight488, 1:800, Jackson ImmunoResearch, USA, 711-485-152; DyLight649, 1:500, Abcam, USA, ab102263) and a Cy3 secondary antibody (Cy3, 1:500, Jackson ImmunoResearch, USA, 115-165-166). Cell nuclei were labeled by 4’,6-Diamidin-2-phenylindol (DAPI) counterstaining (PanReac AppliChem GmbH, Germany, A1001).

#### ChAT labeling

Cervical and lumbar spinal cord sections of 3 months old animals were treated for 15 min with citrate buffer at 95°C and a 1 mg/ml sodium borohydride/PBS solution and subsequently blocked with 10% horse serum for one hour. Thereafter sections were labeled with a ChAT antibody (1:300, Millipore, USA, AB144P). Binding of the primary antibody was visualized using a highly cross-absorbed DyLight secondary antibody (DyLight650, 1:500, Abcam, USA, ab96938). Cell nuclei were labeled by DAPI counterstaining (PanReac AppliChem GmbH, Germany, A1001).

Grey-scale images for quantitative analysis were recorded with an AxioCam MRm camera mounted on a Zeiss Axio Imager.Z1 epifluorescence microscope at 10x magnification. Exposure time and additional setting details were kept constant within the same quantification. ChAT^+^ cells were counted manually (overlapping of ChAT and DAPI staining). For evaluation of TDP-43, GFAP and CD11b expression, regions of interest (ROI) were defined by individual delineation of the whole isocortex, spinal cord, hypothalamus and medulla oblongata. These brain regions were chosen after manual evaluation of TDP-43 expression on sagittal sections. Immunofluorescence signals were measured by adequate thresholding and morphological filtering (size, shape) throughout the ROIs of all images using ImageProPlus Software (Version 6.2). Individual means were determined from three to five sections per animal. Group means were calculated by averaging the individual means. Analyzed parameter were immunoreactive area (IR) in percent of the ROI area and summarized object intensity in a.u./mm^2^.

### Statistics

Data analysis was performed in GraphPad Prism^™^ 4.03 (GraphPad Software Inc., USA). Graphs show group means and standard error of the mean (SEM). The significance level was set at p < 0.05. Group means were compared using Student’s t-test with Welch’s correction or a Two-way analysis of variance (ANOVA) with a subsequent *post-hoc* test. The utilized statistical tests and exact sample numbers are mentioned in the figure legends.

## Results

### hTDP-43 protein is highly expressed in the CNS of TAR6/6 mice

**Fig 1:**
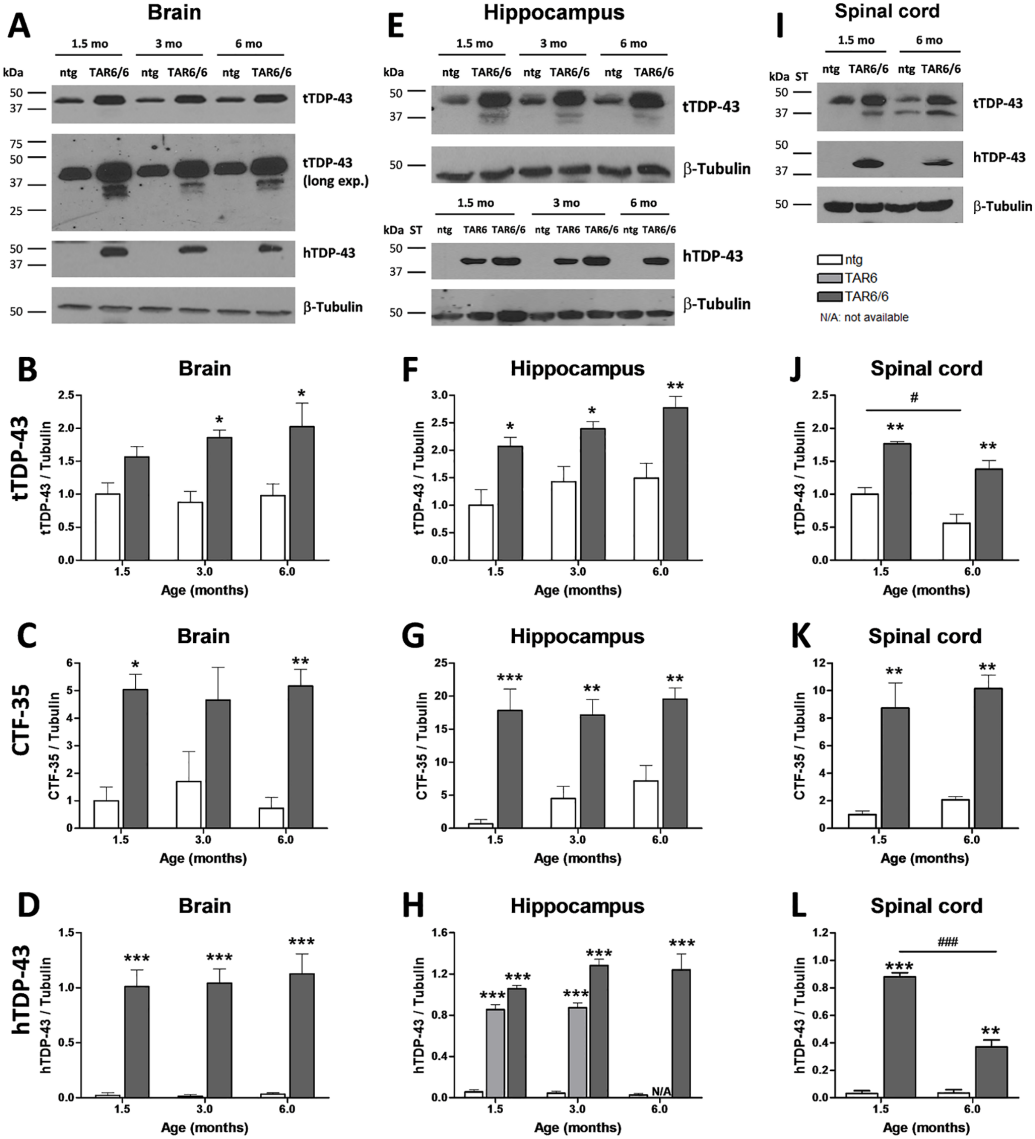
Densitometric analysis of tTDP-43, CTF-35 and hTDP-43 expression in ntg, TAR6 and TAR6/6 mice. (A) Brain homogenates, (E) hippocampal and (I) spinal cord homogenates from ntg, TAR6 and TAR6/6 mice at the age of 1.5, 3 and 6 months were analyzed by Western blotting and probed with the indicated antibodies (long exp. = long time ECL exposure for 30 min). One representative example of 3 is shown. Densitometric analysis of tTDP-43, CTF-35 and hTDP-43 levels normalized to β-tubulin levels of brain (B- D), hippocampal (F-H) and spinal cord (J-L) homogenates. Two-way ANOVA followed by Bonferroni‘s *post-hoc* test. Mean+SEM. *significances between genotypes, ^#^significances between age groups. *p<0.05, **p<0.01, ***p<0.001.

For quantification of total, truncated and human TDP-43 protein levels, Western blot analyses of total brain, hippocampus and spinal cord tissue obtained from 1.5, 3 and 6 months old TAR6/6 and non-transgenic (ntg) littermates were performed (Fig 1A, 1E and 1I).

Total TDP-43 levels (tTDP-43) were analyzed in ntg and TAR6/6 mice (Fig 1B, 1F and 1J). tTDP-43 was approximately 1.5-to 2-fold overexpressed in brain and spinal cord samples and 2- to 3-fold increased in hippocampus homogenates of TAR6/6 mice. While tTDP-43 levels did not significantly alter over age in the brain and hippocampus, levels were reduced in ntg spinal cord samples at an age of 6 months. Furthermore, the 35 kDa C-terminally truncated fragment (CTF-35) of TDP-43 was found in TAR6/6 samples and 20-fold increased in the hippocampus, 10-fold in the spinal cord and 5-fold in whole brain samples (Fig 1C, 1G and 1K).

In addition to tTDP-43 and CTF-35, densitometric analyses of immunoreactive signals revealed high hTDP-43 levels in the central nervous system already at the age of 1.5 months in TAR6/6 mice (Fig 1D, 1H and 1L). hTDP-43 levels stayed unchanged over age in the whole brain and hippocampus, while levels progressively decreased in the spinal cord of TAR6/6 mice (Fig 1L). As an exemplary analysis, hTDP-43 was also investigated in hippocampus of 1.5 and 3 months old hemizygous TAR6 mice (Fig 1H). An overexpression of hTDP-43 could be detected in hemizygous TAR6 but it was not as strong as in homozygous TAR6/6 mice. Interestingly, even after 2 hours of exposure, the 35 kDa CTF could not be detected by the hTDP-43 antibody (Fig 1A, 1E and 1I).

**Fig 2:**
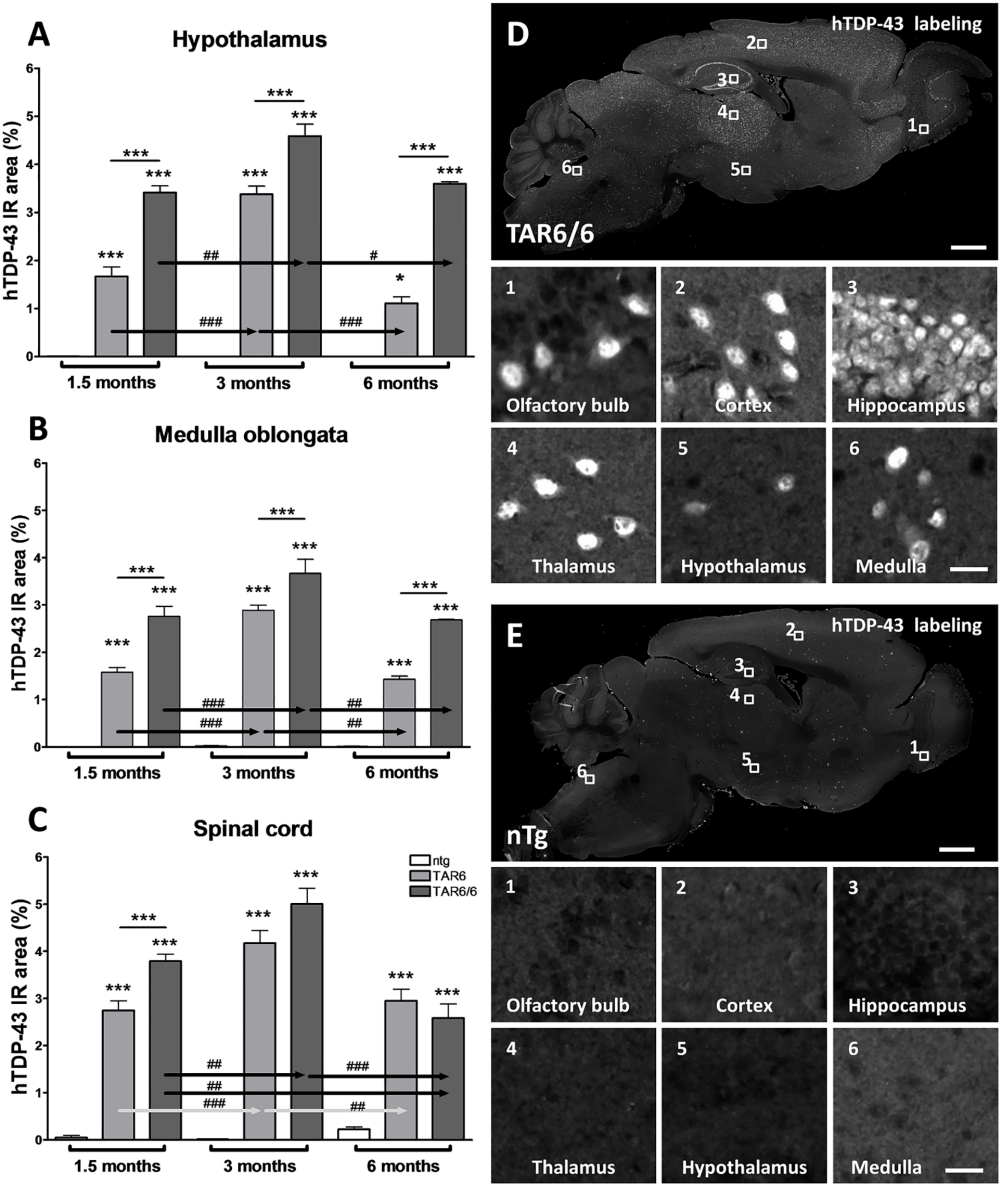
Quantification of hTDP-43 expression in ntg, TAR6 and TAR6/6 mice. Quantitative hTDP-43 expression in hypothalamus (A), medulla oblongata (B) and spinal cord (C) analyzed by immunofluorescent labeling of CNS samples. (D, E) Representative overview image and magnifications of hTDP-43 labeling of a brain section (olfactory bulb (1), cortex (2), hippocampus (3), thalamus (4), hypothalamus (5) and medulla oblongata (6)) of 3 months old TAR6/6 (D) and ntg (E) mice. Scale bars: Overview images = 1000 μm; magnification images = 20 μm. (A, B) 1.5 months: ntg: n = 4; TAR6: n = 10; TAR6/6: n = 5; 3 months: ntg: n = 5; TAR6: n = 8; TAR6/6: n = 5; 6 months: ntg: n = 3; TAR6: n = 3; TAR6/6: n = 3. (C) n as in A, B exempt: 1.5 months: ntg: n = 3; TAR6: n = 11. (A-C) Two-way ANOVA followed by Bonferroni‘s *post-hoc* test. Mean+SEM. *significances between genotypes, ^#^significances between age groups. *p<0.05, **p<0.01, ***p<0.001.

Furthermore, histological analysis of hTDP-43 expression levels in the hypothalamus, medulla oblongata and spinal cord grey matter, including anterior and posterior horn areas, revealed a high expression of hTDP-43 protein in TAR6, TAR6/6 and ntg mice already at the age of 1.5 months (Fig 2A–2E). We observed even higher expression of hTDP-43 protein in 3 months old TAR6 and TAR6/6 mice compared to 1.5 months old animals. Further aging of TAR6 and TAR6/6 animals caused a significant hTDP-43 decrease in both genotypes compared to 3 months old animal. In general, TAR6/6 animals expressed significantly more hTDP-43 than TAR6 as shown by an increased immunoreactive area (IR). Analysis of the sum object intensity resulted in very similar results exempt in the hypothalamus of 6 months old TAR6 and TAR6/6 mice, were the signal was strongly reduced compared to younger mice (S1 Fig).

### Cytoplasmic localization of hTDP-43 is increased in TAR6/6 mice

**Fig 3:**
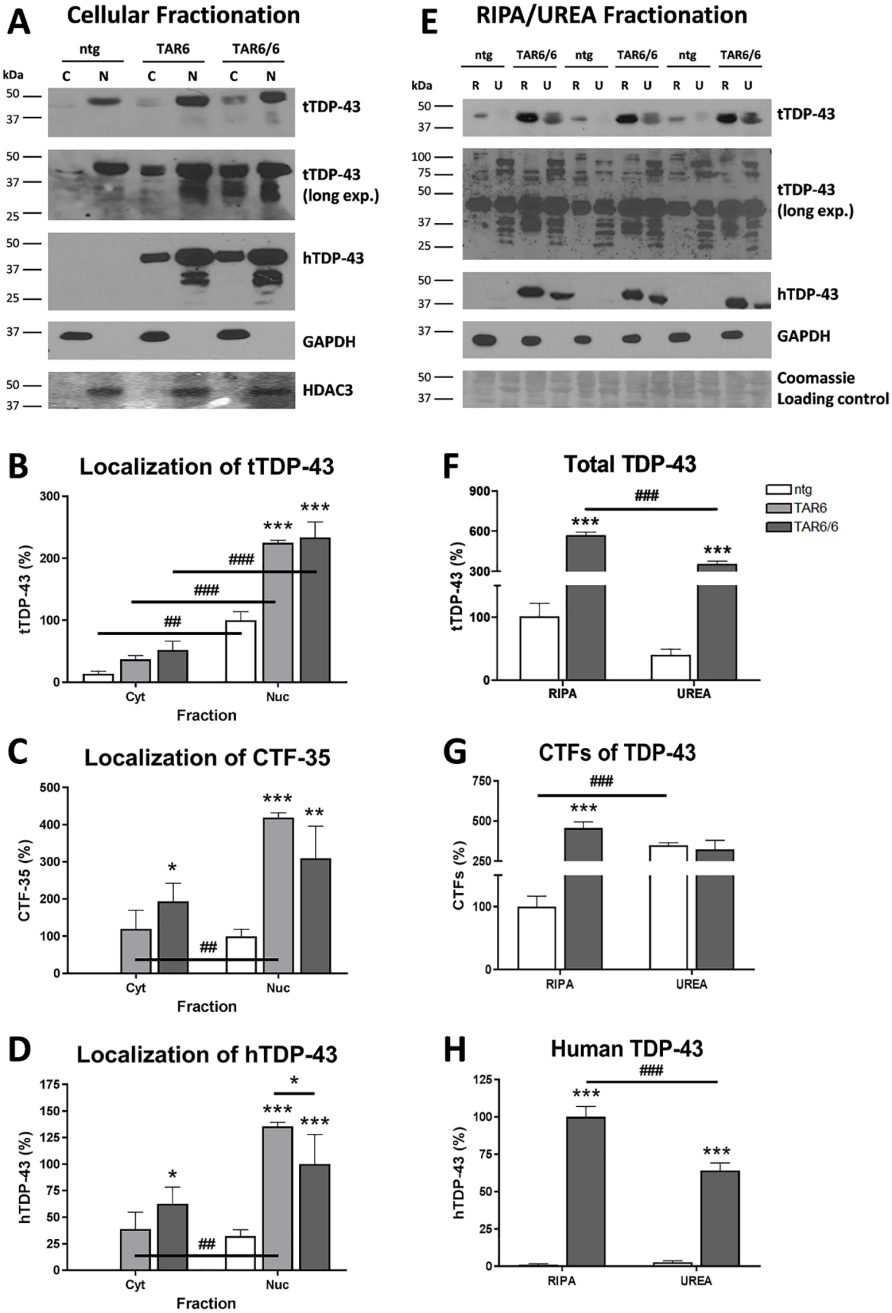
Posttranslational changes in TAR6/6 mice. (A-D) Localization of tTDP-43, CTF-35 and hTDP-43 after cellular fractionation of 3 months old TAR6/6 mice. (A) Cell fractions of midbrain and cortex samples of 3 months old ntg, TAR6 and TAR6/6 mice were analyzed by Western blotting and probed with the indicated antibodies (GAPDH as cytoplasmic and HDAC3 as nuclear marker; long exp. = long time ECL exposure for 30 min). One representative example of 4 is shown (C: cytoplasm, N: nucleus). (B- D) Densitometric analysis of tTDP-43, CTF-35 and hTDP-43 levels in cell fractions (cyt: cytoplasm, nuc: nucleus). Nuclear tTDP-43 and CTF-35 levels of ntg and hTDP-43 of TAR6/6 were set as 100%. (E-H) Soluble and insoluble TDP-43 protein levels in brains of 3 months old TAR6/6 mice. Brain samples were separated into RIPA soluble (R) and RIPA insoluble / UREA fractions (U). Three representative examples of 5 are shown. Fractions were analyzed by Western blotting and probed with the indicated antibodies (long exp. = long time ECL exposure for 30 min). Coomassie Blue staining was performed as loading control for UREA fraction. (F-H) Densitometric analysis of tTDP-43, CTFs and hTDP-43 levels in RIPA and UREA fractions. For tTDP-43 and CTF evaluation, RIPA fraction of ntg was set as 100%, whereas for hTDP-43 analysis, RIPA sample of TAR6/6 was set as 100%. (B-D; F-H) Two-way ANOVA followed by Bonferroni‘s *post-hoc* test. Mean+SEM. *significances between genotypes, ^#^significances between fractions. *p<0.05, **p<0.01, ***p<0.001.

To investigate the cellular localization of TDP-43 protein, midbrain and cortex tissue of 3 months old ntg, TAR6 and TAR6/6 mice was separated into cytoplasmic and nuclear fractions and analyzed by Western blot (Fig 3A–3D). tTDP-43 was predominantly localized in the nucleus in all three genotypes (Fig 3A) and significantly enriched in TAR6 and TAR6/6 samples (Fig 3B). Moreover, after long-time ECL exposure of the tTDP-43 blot, CTFs could not only be detected in the nuclear fraction of TAR6 and TAR6/6 mice but was also significantly increased in the cytosolic fraction of TAR6/6 mice (Fig 3A (long exp.), C). In addition, hTDP-43 staining could confirm that overexpressed TDP-43 accumulates in the cytoplasm of TAR6/6 mice and that nuclear CTFs are produced by hTDP-43 (Fig 3A and 3D).

### Insoluble TDP-43 accumulates in TAR6/6 mice

Furthermore, it was analyzed whether overexpression of hTDP-43 enhances insolubility of TDP-43. For this purpose, brain samples of 3 months old TAR6/6 and ntg mice were separated into RIPA soluble and RIPA insoluble (UREA) fractions and analyzed by Western blot using antibodies against tTDP-43 and hTDP-43 (Fig 3E–3H). In the RIPA soluble fraction, a signal for total full-length TDP-43 in TAR6/6 and ntg samples (Fig 3F) and after long-time ECL exposure a specific 35 kDa CTF in the TAR6/6 samples could be detected (Fig 3G). Additionally, a signal for human full length TDP-43 could be detected in TAR6/6 but not in ntg mice (Fig 3H). Furthermore, TAR6/6 mice showed high levels of tTDP-43 (Fig 3F) and hTDP-43 (Fig 3H) in the insoluble UREA fraction. Interestingly, no differences in CTF levels between TAR6/6 and ntg in the UREA fraction by using antibodies against tTDP-43 (Fig 3G) and hTDP-43 (data not shown) could be detected. This result suggests that fragment bands of the UREA fraction shown by tTDP-43 labeling are non-specific and do not depend on the genotype.

### Co-localization of human and total TDP-43 by immunofluorescent labeling

**Fig 4:**
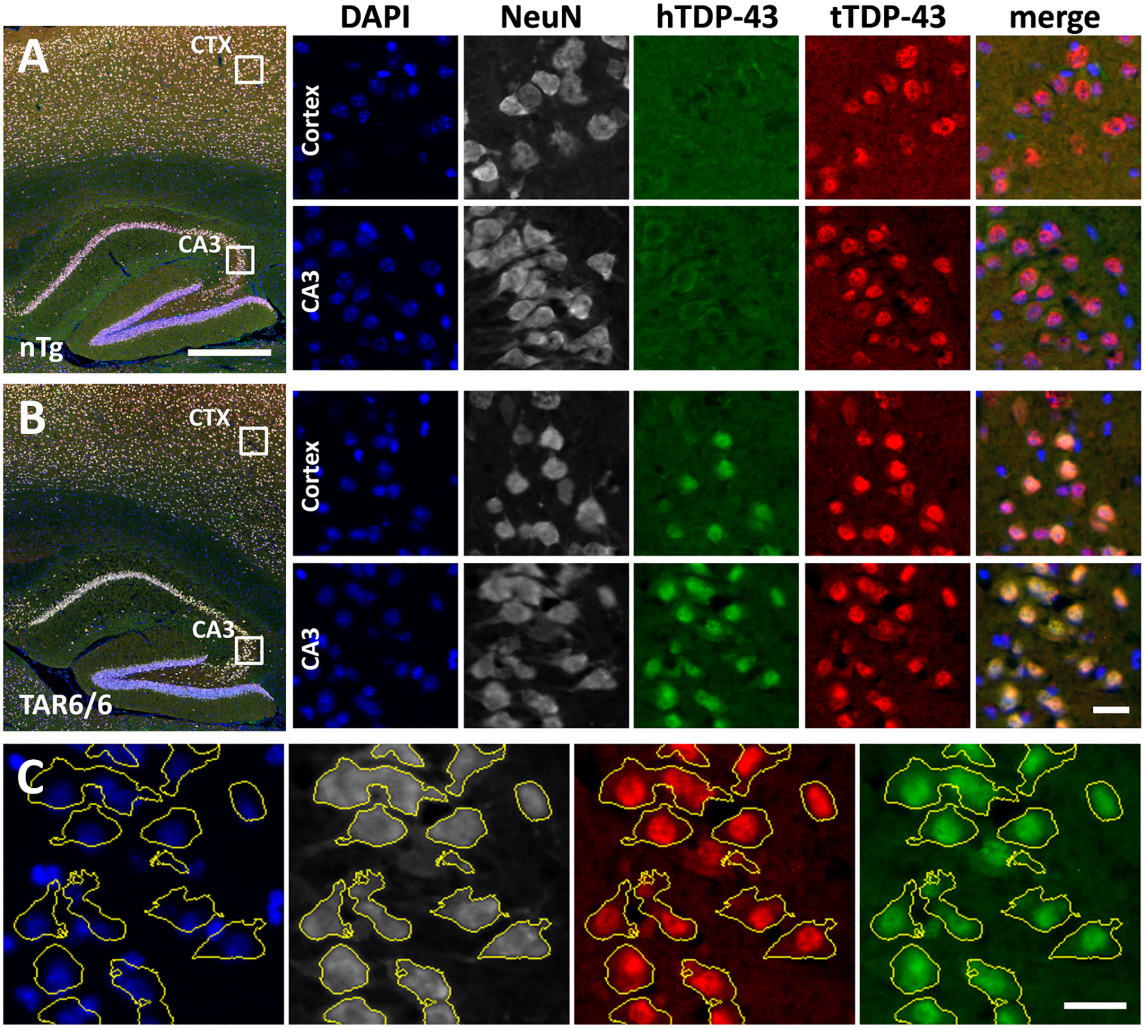
Immunofluorescent double-labeling of human and total TDP-43. Cortical and hippocampal tissue of three-months old ntg (A) and TAR6/6 (B) mice was labeled with antibodies against human TDP-43 (green) and total TDP-43 (red). Tissue was additionally labeled with NeuN antibody (white) and DAPI (blue) to visualize neuronal somata and nuclei, respectively. The merged images in the right column show human TDP-43, total TDP-43 and DAPI labeling. (C) The preferred nuclear localization of both human TDP-43 and total TDP-43 is obvious when the outlines of NeuN-positive somata are projected on the TDP-43 channels. Scale bars: Overview images = 500 μm; magnification images = 20 μm; (C) = 20 μm.

In order to visualize the subcellular localization of human and total TDP-43 protein as observed by cell fractionation and Western blotting, co-labeling of both proteins by immunofluorescence was performed (Fig 4) in 3 months old ntg (Fig 4A) and TAR6/6 (Fig 4B) mice. High magnification images of the cortex and hippocampus show high immunofluorescence of human and total TDP-43 in the nucleus and only minor immunofluorescence in the cytoplasm of TAR6/6 mice, consistent with the biochemical analysis. In the cortex, more cells were positive for total TDP-43 than for human TDP-43. Human TDP-43 immunoreactivity was absent from ntg brain tissue (Fig 4A). The histological results generally support the biochemical findings on expression and subcellular localization of TDP-43 protein.

### Neuroinflammation in TAR6/6 mice

**Fig 5:**
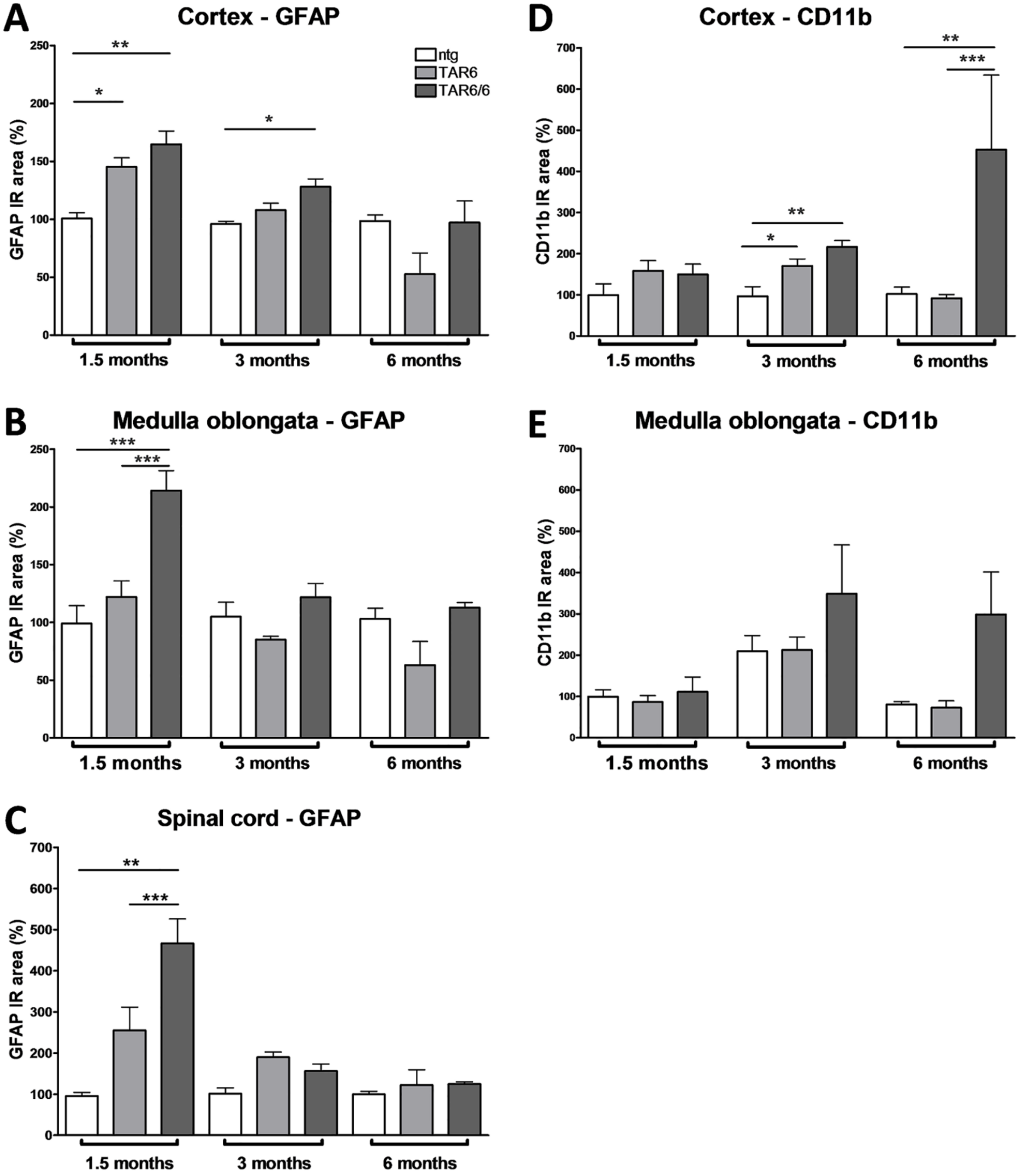
Quantification of GFAP and CD11b expression in the central nervous system of ntg, TAR6 and TAR6/6 mice. (A-C) GFAP expression levels labeling reactive astrocytes in the cortex (A), medulla oblongata (B) and spinal cord (C); and CD11b expression levels labeling reactive microglia in the cortex (D) and medulla oblongata (E) as IR area in percent. (A) 1.5 months: ntg: n = 4; TAR6: n = 9; TAR6/6: n = 5; 3 months: ntg: n = 4; TAR6: n = 9; TAR6/6: n = 5; 6 months: ntg: n = 3; TAR6: n = 3; TAR6/6: n = 3. (D) n as in A except: 1.5 months: TAR6: n = 10; 3 months: ntg: n = 5; TAR6: n = 8. (B, E) n as in D except: 3 months: TAR6/6: n = 7. C) n as in B except: 1.5 months: TAR6: n = 5; TAR6/6: n = 3; 3 months: ntg: n = 3; TAR6: n = 7; TAR6/6: n = 4. (A-E) Two-way ANOVA followed by Bonferroni‘s *post-hoc* test. Mean+SEM. *significances between genotypes. *p<0.05, **p<0.01, ***p<0.001.

Neuroinflammation has been described as one of the major hallmarks of neurodegenerative diseases (for review see [24]) and also linked to ALS/FTLD [25,26]. For this reason, IR area of glial fibrillary acidic protein (GFAP) as marker for reactive astrocytes was quantified over age. Significantly increased levels in TAR6/6 and TAR6 mice compared to ntg animals could be found in the cortex (Fig 5A), medulla oblongata (Fig 5B) and spinal cord (Fig 5C) at the age of 1.5 months as well as in the cortex of TAR6/6 mice at an age of 3 months (Fig 5A). Analysis of GFAP at the age of 6 months revealed no significant differences between genotypes. TAR6 mice followed a similar, but weaker expression pattern as TAR6/6 (Fig 5A, 5B and 5C). Labeling of cortical tissue with cluster of differentiation 11b (CD11b) as marker for activated microglia revealed a slight increase at 3 months of age and a severe microglia activation in 6 months old TAR6/6 mice compared to ntg and TAR6 animals (Fig 5D). In the medulla oblongata the same trend was detected (Fig 5E). Representative images of labelings are shown in S2 Fig.

### Neuronal loss in the spinal cord of TAR6/6 mice

**Fig 6:**
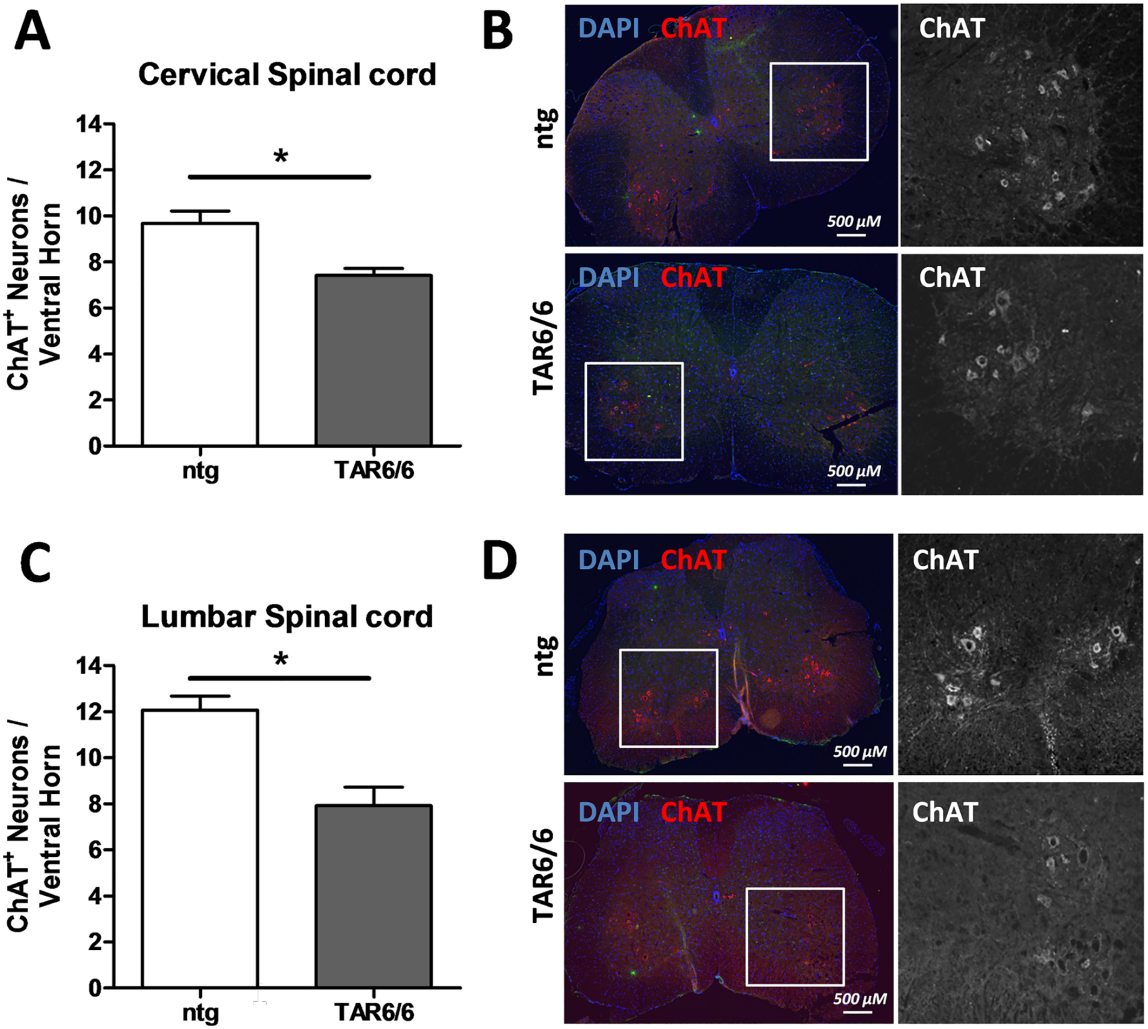
Quantification of neuron loss in the spinal cord of 3 months old TAR6/6 mice. (A, C) Number of ChAT^+^ neurons in the ventral horn of the cervical (A) and lumbar (C) spinal cord grey matter. (B, D) Representative images of ChAT labeling in the cervical (B) and lumbar (D) spinal cord of 3 months old TAR6/6 mice compared to ntg animals. Nuclei of cells were stained by DAPI. (A) ntg: n = 4, TAR6/6: n = 3. (C) ntg: n = 3, TAR6/6: n = 3. Unpaired Student’s t-test followed by Welch’s correction. Mean+SEM. *p<0.05.

Since ALS pathology is accompanied by loss of motoneurons in the spinal cord, the number of choline acetyltransferase (ChAT) positive neurons was counted in cervical and lumbar spinal cord regions of 3 months old ntg and TAR6/6 mice (Fig 6A–6D). In both regions ChAT positive neurons were significantly decreased in the ventral horn indicating degeneration of motoneurons in TAR6/6 mice.

### TAR6/6 mice develop severe motor deficits

**Fig 7:**
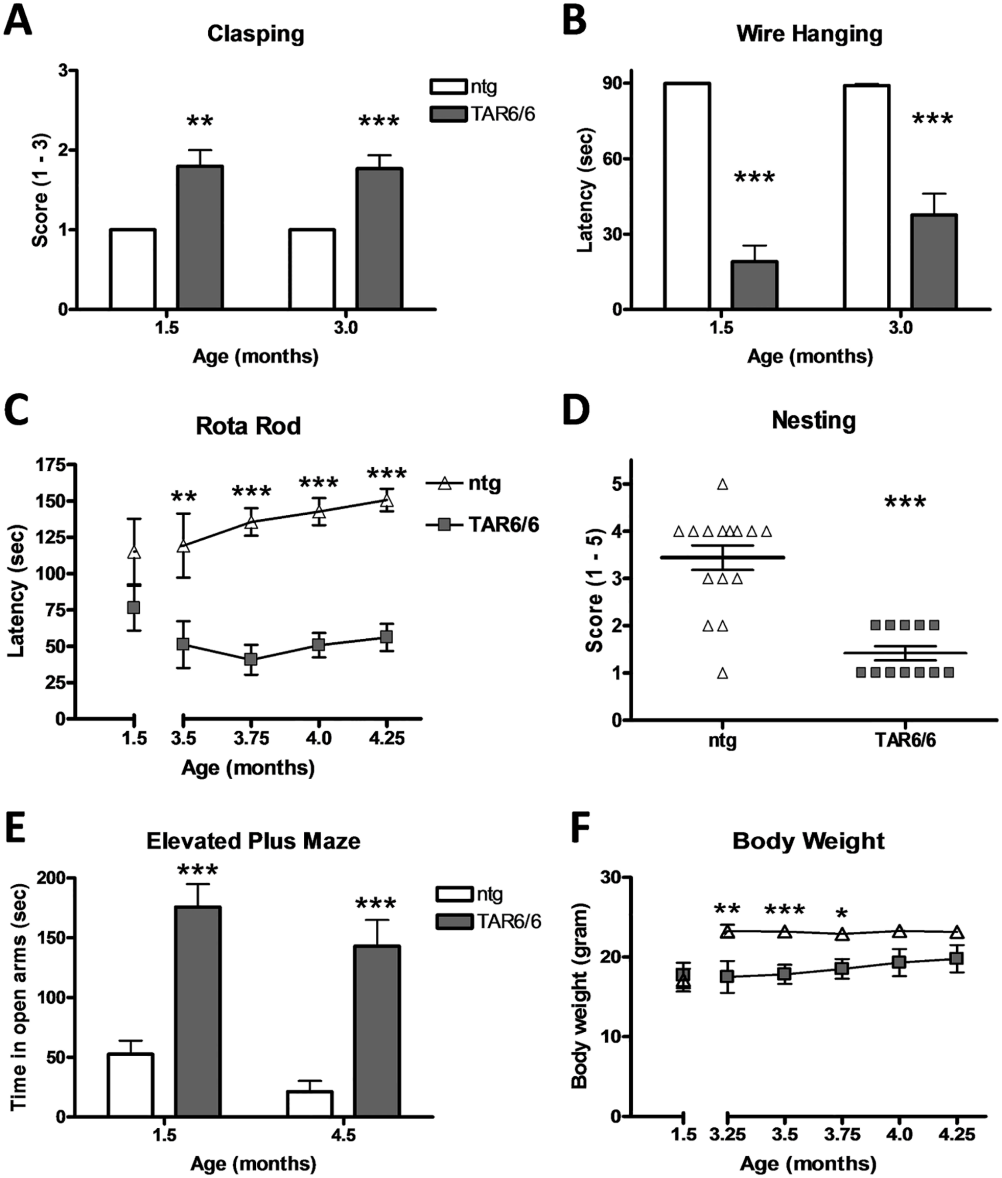
Motor impairments and reduced anxiety in TAR6/6 mice. (A) Clasping behavior scored from 1–3 of 1.5 and 3 months old TAR6/6 mice compared to ntg littermates (score of 1: normal clasping). (B) Latency to fall in the wire suspension test in seconds of 1.5 and 3 months old TAR6/6 mice compared to ntg littermates. (C) Latency to fall from the rotating rod in the Rota Rod test of 1.5 to 4.25 months old TAR6/6 mice compared to ntg littermates. (D) Nesting behavior scored from 1–5 of 3 months old TAR6/6 mice compared to ntg littermates (high score: perfect nest). (E) Time in seconds of 1.5 and 4.5 months old TAR6/6 mice spent in the open arms of the elevated plus maze compared to ntg littermates. F: Body weight in gram of 1.5 to 4.25 months old TAR6/6 mice compared to ntg mice of the same age. (A, B) 1.5 months: TAR6/6: n = 5, ntg: n = 7; 3 months: TAR6/6: n = 13, ntg: n = 16. (C) 1.5 months: TAR6/6: n = 5, ntg: n = 7; 3.5 months: TAR6/6: n = 8, ntg: n = 7; 3.75 months: TAR6/6: n = 12, ntg: n = 16; 4 months: TAR6/6: n = 10, ntg: n = 16; 4.25 months: TAR6/6: n = 9, ntg: n = 16. (D) TAR6/6: n = 16, ntg: n = 12. E: 1.5 months: TAR6/6: n = 6, ntg: n = 4; 4.5 months: TAR6/6: n = 8, ntg: n = 16. (F) 1.5 months: TAR6/6: n = 5, ntg: n = 7; 3.25 months: TAR6/6: n = 7, ntg: n = 12; 3.5 months: TAR6/6: n = 12, ntg: n = 18; 3.75 months: TAR6/6: n = 10, ntg: n = 18; 4.0 months: TAR6/6: n = 8, ntg: n = 18; 4.25 months: TAR6/6: n = 8, ntg: n = 18. (A-C, E, F). Two-way ANOVA followed by Bonferroni‘s *post-hoc* test. D: Mann Whitney U-test. A,B,E: Mean+SEM; C,D,F: Mean±SEM. *p<0.05, **p<0.01, ***p<0.001.

TAR6/6 mice were tested for hind limb reflexes at the age of 1.5 and 3 months. Our results show that already at the age of 1.5 months TAR6/6 mice reached a significantly higher clasping score compared to ntg animals (Fig 7A). This effect was also prominent at the age of 3 months. Further analyses of TAR6/6 mice for motor abilities show that 1.5 months old TAR6/6 mice presented severe neuromuscular deficits as revealed by the significantly decreased hanging time in the wire suspension test (Fig 7B). Analysis of 3 months old animals in this test confirmed the results obtained with young animals. Thus, TAR6/6 mice exhibit highly decreased muscle strength. Additional analyses of TAR6/6 mice in the Rota Rod test over age showed a strongly decreased latency to fall from the rod starting at the age of 3.5 months (Fig 7C). Even though mice showed an early muscle weakness in the wire suspension test, the first deficits in the Rota Rod were only detectable at the age of 3.5 months (Fig 7B vs. 7C). Next to these explicit motor assays, TAR6/6 mice were analyzed in the nest building test. TAR6/6 mice reached a significantly lower nesting score at the age of 3 months compared to ntg littermates (Fig 7D).

### TAR6/6 mice exhibit reduced anxiety

To test TAR6/6 mice for anxiety, mice were assessed in the elevated plus maze test. 1.5 and 4.5 months old TAR6/6 mice spent significantly more time in the open arms of the elevated plus maze compared to ntg littermates (Fig 7E) indicating reduced anxiety levels of TAR6/6 mice.

### Decreased body weight of TAR6/6

To validate that motor deficits are not caused by obesity of TAR6/6 mice, the first cohort of animals was weight once at 1.5 months of age and a second cohort from the age of 3.25 to 4.25 months. Analysis by Two-way ANOVA revealed a comparable body weight at young age but a significantly decreased body weight of TAR6/6 mice at the age of 3.25 to 3.75 months. With increasing age, this difference attenuates to comparable levels (Fig 7F).

Analysis of up to 12 months old hemizygous TAR6 mice in a similar test battery did not reveal significant behavioral abnormalities compared to ntg littermates (S3 Fig).

In summary, we observed a strong hTDP-43 overexpression followed by ALS/FTLD pathologies, such as enriched cytoplasmic and insoluble TDP-43, neuroinflammation as revealed by increased astrogliosis and microgliosis, loss of motor neurons in the spinal cord as well as pronounced motor abnormalities in homozygous TAR6/6 mice (Table 1).

**Table 1:** Summary of pathological features of TAR6/6 mice.

		1.5 months	3–4.5 months	6 months
**Cellular pathology**	Cytoplasmic hTDP-43	N/A	↑	N/A
	Nuclear hTDP-43	N/A	↑	N/A
	Insoluble hTDP-43	N/A	↑	N/A
	CTFs	↑	↑	↑
**GFAP**	Cortex	↑	↑	N.S.
	Medulla oblongata	↑	N.S.	N.S.
	Spinal cord	↑	N.S.	N.S.
**CD11b**	Cortex	N.S.	↑	↑
	Medulla oblongata	N.S.	N.S.	N.S.
**ChAT**	Spinal cord	N/A	↓	N/A
**Motor Tests**	Clasping score	↑	↑	N/A
	Wire hanging time	↓	↓	N/A
	Rota Rod performance	↓	↓	N/A
	Nest building ability	N/A	↓	N/A
**Anxiety Test**	Time in open arms (Elevated Plus Maze)	↑	↑	N/A

Results of cellular pathology, neuroinflammation, neuron loss as well as motor and anxiety tests in TAR6/6 mice compared to ntg mice at an age of 1.5, 3–4.5 and 6 months. N/A: not available. N.S.: not significant. ↑: increase, ↓: decrease.

## Discussion

To represent a suitable new transgenic animal model for compound testing it is essential that the model ensures a high construct and face validity. Since TDP-43 is well known to be a major factor in the development of human ALS and the here presented TAR6/6 mouse model overexpresses human wild type TDP-43 protein [17], the construct validity is well given. With the present data, the face validity of the TAR6/6 model was analyzed in detail.

Biochemical analysis of hTDP-43 levels showed a strong expression in the total brain, hippocampus and spinal cord of TAR6/6 mice and a slightly weaker expression in TAR6 mice. The expression pattern of hTDP-43 is comparable with evaluated tTDP-43 levels which are 1.5 to 2.5 fold increased depending on the tissue. The overexpression might seem to be rather weak but Ayala and colleagues previously showed that endogenous TDP-43 levels can be downregulated by binding of overexpressed TDP-43 in the 3’UTR region of the endogenous mRNA [27]. We hypothesize that endogenous TDP-43 is widely depleted and the detected tTDP-43 levels mainly consist of hTDP-43. In addition to increased tTDP-43 levels, the 35 kDa C-terminally truncated fragment (CTF-35) of TDP-43 that is clearly detectable in human FTLD samples [28], could be found in TAR6/6 mice. This result is consistent with a previous analysis of FTLD and ALS cases and therefore presenting the biochemical similarity of TAR6/6 mice and human TDP-43 related diseases [29]. On the other hand, the 25 kDa fragment, which has also been associated with FTLD [28], could not be detected in transgenic animals assuming that the model mimics rather ALS than FTLD pathology where fragments are less common.

Overall histological analyses showed similar expression levels of TDP-43 in different brain regions. We observed an increase of hTDP-43 in the hypothalamus, medulla oblongata and spinal cord up to an age of 3 months, followed by a slight decrease at the age of 6 months which was observed by Western blot in the spinal cord but not in brain and hippocampal tissue. The decrease in TDP-43 expression at higher age might be caused by a loss of hTDP-43 expressing neurons. The reduced ChAT labeling in the spinal cord of TAR6/6 samples thus indicates an increased motoneuron death which is accompanied by neuronal loss of motor cortex layer V and anterior horn neurons detected by analyzing Cresyl violet staining as described by Wils *et al*. [17]. Furthermore, CD11b labeling of activated microglia is slightly increased in the cortex at an age of 3 months and highly increased in 6 months old TAR6/6 mice supporting the assumption that neurons start to degenerate at 3 months. On the contrary, GFAP labeling of activated astrocytes was strongly increased at an age of 1.5 months in the cortex, medulla oblongata and spinal cord and decreased over age in TAR6/6 mice while only slight activation of astroglia and microglia was observed in the cortex of 1.5 and 3 months old TAR6 mice, respectively. In 2010, Wils *et al*. analyzed inflammation processes in the TAR6/6 mice by GFAP and Iba-1 staining. The group also revealed activation of microglia in 6 months old animals but additionally activation of astrocytes by GFAP at the age of 6 months in the motor cortex layer V and anterior horn of the spinal cord [17]. The observed astrogliosis and microgliosis in different brain regions of TAR6/6 mice provides evidence for a similar neuroinflammatory pathology compared to another TDP-43 transgenic mouse [30], the SOD1 mouse model [31], human ALS cases [32] or human FTLD with mutated progranulin [33]. Neuroinflammation is thus a mutual neuropathological feature of ALS/FTLD occurring in the most common types of genetically altered ALS models.

Although expression levels of hTDP-43 and inflammation processes in hemizygous TAR6 and homozygous TAR6/6 mice differ, our biochemical and histological data consistently indicate that TDP-43 is strongly enriched in the nucleus whereas immunoreactivity is much lower in the cytoplasm. Of note, we did not detect a shift of subcellular hTDP-43 from the nucleus to the cytoplasm. On the contrary, the genotypes display significantly increased nuclear hTDP-43 although in human ALS/FTLD samples the absence of nuclear TDP-43 has been described and correlated to crucial pathological features of ALS/FTLD [34]. Furthermore, 35 kDa CTFs can be found in the nuclear fractions by staining for tTDP-43 and hTDP-43 and in the cytoplasmic fraction by staining for tTDP-43. The 35 kDa CTF is known to be cleaved by caspase-3 [35] at cleavage site DETD^89^ [28,36] which has been shown to be activated in TAR6/6 mice by Wils *et al*. [17]. The fragment is thought to be involved in the formation of cytoplasmic inclusion bodies as well as pulling the functional full-length TDP-43 out of the nucleus, which is then additionally integrated in the cytosolic inclusion bodies [36]. One interesting difference between TAR6 and TAR6/6 mice is the presence of the 35 kDa fragment only in the cytoplasm of TAR6/6 mice. This cytoplasmic CTF might cause the described pathogenic effects of full-length TDP-43 and contribute to the development of phenotypic symptoms of TAR6/6 mice.

In another experiment, the influence of overexpressed hTDP-43 on the solubility of TDP-43 was investigated. In 3 months old TAR6/6 mice, tTDP-43 as well as hTDP-43 was highly enriched in the RIPA insoluble (UREA) fraction. Thus, insoluble TDP-43 was accumulated in brains of TAR6/6 animals and mimics one of the well described hallmarks of human ALS/FTLD pathology [37]. The 35 kDa CTF has been described to induce aggregation because of its own insolubility [36] and is supposed to be concentrated in the insoluble UREA sample. However, no differences in the pattern of the CTF bands between ntg and TAR6/6 mice in the UREA fraction could be observed in the here presented study. In contrast, the 35 kDa CTF was significantly increased in the soluble RIPA fraction. This unusual fractionation might result in a pathology start caused by high hTDP-43 levels in the nucleus as well as the cytoplasm at 3 months of age. As a result of toxic concentrations, full-length TDP-43 would be processed and cleaved into CTFs which might still be soluble but already induce insolubility of full-length TDP-43 and would become insoluble at a later age. To clarify this hypothesis, experiments with older animals are needed in future.

To demonstrate a similarity of behavioral deficits between TAR6/6 mice and ALS patients, several specific tests were performed. Mice not only presented altered reflexes and muscle weakness but also strong deficits in motor coordination as shown with the Rota Rod test. These results are comparable with muscle weakness, gait disturbance, coordination problems as well as weakness in arm and leg muscles described in ALS patients (reviewed in [38]). Besides these typical motor disturbances TAR6/6 mice exhibited decreased anxiety. A similar phenotype could also be observed in a conditional TDP-43 transgenic mouse model [39] but for many published TDP-43 transgenic mouse models, the anxiety status is not described yet [15,18,40,41]. It is thus not possible to evaluate, if this phenotype is typical in such models although increased anxiety levels are common in ALS patients [42,43]. Nest building in rodents is a multifaceted behavior conducted for thermoregulation and protection of offspring and requires fine motor skills [44]. Reduced nest building behavior can thus be indicative for a general reduction of well-being. When observed in combination with motor deficits as observed in TAR6/6 mice, deficits of fine motor skills of the fore paws and orofacial motor abilities are most likely. Additionally, we could already show that TAR6/6 mice display severe gnawing deficits as analyzed with the pasta gnawing test [23]. These data can be related to dysphagia and other bulbar deficits that are well known in ALS/FTLD patients [45–47] and support the results of the nest building test. The analysis of learning and memory deficits in TAR6/6 mice would also be of great value, since many patients show such symptoms that even correlate with hippocampal volume [48] and a shorter survival [49]. Many learning tests for mice such as the Morris water maze or two choice swim test require good motor performance since animals often have to navigate or even swim. Due to strong motor deficits of TAR6/6 mice the analysis of learning would require the use of a learning test that is not solely dependent on motor performance, e.g. the poke hole test, radial arm maze or Barnes maze. Furthermore, ALS patients often show increased aggression [42] or impairments to set shifting behavior from one response to another [50]. It would therefore also add value to this ALS model to analyze TAR6/6 mice in corresponding behavioral tests.

TAR4/4 mice, which were in detail characterized by Wils and colleagues, have an average survival of only 24 days while TAR6/6 mice live on average 6–7 months [17]. This extended life span strongly improves the usability of TAR6/6 mice for the analysis of new compounds against ALS/FTLD symptoms and pathologies, since these mice present a reasonable time window for a compound to be effective. A shorter life span could mask a drug effect while a longer life span would not properly mimic the severe progression of human ALS symptoms. Additionally, some of the here and previously described behavioral deficits of TAR6/6 mice exhibit a delayed onset, such as the body weight, Rota Rod and pasta gnawing test [23], allowing a treatment start before onset of these symptoms and thus for the analysis of preventive compound effects.

In summary, our study confirms results obtained by Wils et al., such as expression of hTDP-43 in the CNS, increased microgliosis and motor deficits evaluated by the Rota Rod and clasping behavior [17]. In addition, we show that TAR6/6 mice exhibit further prominent ALS/FTLD pathologies, such as cytoplasmic and insoluble TDP-43, CTFs of TDP-43 and loss of ChAT^+^ neurons in the spinal cord (Table 1). TAR6/6 mice thus represent not only pathological TDP-43 expression but also disease-relevant posttranslational changes, substantiating the face validity of this animal model. Combining the results of already published data and the here presented results it can be assumed that TAR6/6 mice represent a valid ALS/FTLD model, well-suitable for preclinical compound tests.

## Conclusions

In summary, our results show that TAR6/6 mice are a valid ALS/FTLD model to mimic prominent pathologies of these devastating neurodegenerative diseases. TAR6/6 mice are therefore an adequate model to test new compounds against ALS/FTLD that target TDP-43 overexpression, fragmentation, insolubility, neuroinflammation, motoneuron loss or other TDP-43 downstream signaling pathways.

## Supporting information

S1 Fig (A-C) Quantification of human TDP-43 expression in 1.5, 3 and 6 months old ntg, TAR6 and TAR6/6 mice. Sum object intensity of hTDP-43 expression in hypothalamus (A), medulla oblongata (B) and spinal cord (C) analyzed by immunofluorescent labeling of CNS samples. (A, B) 1.5 months: ntg: n = 4; TAR6: n = 10; TAR6/6: n = 5; 3 months: ntg: n = 5; TAR6: n = 8; TAR6/6: n = 5; 6 months: ntg: n = 3; TAR6: n = 3; TAR6/6: n = 3. (C) n as in A, B exempt: 1.5 months: ntg: n = 3; TAR6: n = 11. (A-C) Two-way ANOVA followed by Bonferroni‘s post-hoc test. Mean+SEM. *significances between genotypes, #significances between age groups. *p<0.05, **p<0.01, ***p<0.001.
(PDF)

S2 Fig GFAP and CD11b labelling in ntg and TAR6/6 mice. (A) Cortical, (B) medulla oblongata, (C) spinal cord GFAP expression levels labelling reactive astrocytes and (D) CD11b expression levels labeling reactive microglia. Representative images of 1.5 months old (A, B, C) and 6 months old (D) animals. Cell nuclei are stained by DAPI.
(PDF)

S3 Fig Motor performance and anxiety levels in hemizygous TAR6 mice compared to non-transgenic littermates. (A) Clasping behavior scored from 1–3 of 1.5 months old TAR6 mice (score of 1: normal clasping). (B) Latency to fall in the wire suspension test in seconds of 1.5, 9 and 12 months old TAR6 mice. (C) Latency to fall from the rotating rod in the Rota Rod test of 1.5 months old TAR6 mice. (D) Nesting behavior score of 1.5 months old TAR6 and TAR6/6 mice (high score: perfect nest). (E) Time in seconds 1.5 months old TAR6 mice spent in the open arms of the elevated plus maze. (F) Body weight in gram of 1.5, 9 and 12 months old TAR6 mice. (A, C) TAR6: n = 11, ntg: n = 7; (E) TAR6: n = 11, ntg: n = 6. (D) TAR6: n = 9, ntg: n = 4, TAR6/6: n = 4. (B, F) 1.5 months: TAR6: n = 11, ntg: n = 7; 9 months: TAR6: n = 15, ntg: n = 12; 12 months: TAR6: n = 15, ntg: n = 13; Mean+SEM. A: One sample t-test; C-E: unpaired t-test. B,F: Two-way ANOVA; A-F: non significant (p>0.05).
(PDF)

S1 Table Raw data of all quantifications presented in the manuscript. (PDF)

## Abbreviations

AAALAC: Association for Assessment and Accreditation of Laboratory Animal Care
ALS: Amyotrophic lateral sclerosis
ANOVA: Analysis of variance
C or cyt: cytoplasmic
CD11b: Cluster of Differentiation 11b
ChAT: Choline acetyltransferase
CTF: C-terminal fragment
DAPI: 4’,6-Diamidin-2-phenylindol
FTLD-U/ FTLD-TDP: frontotemporal lobar degeneration with ubiquitin-positive inclusions
GAPDH: Glycerinaldehyd-3-phosphat-Dehydrogenase
GFAP: Glial fibrillary acidic protein
IR: immunoreactive
N or nuc: nuclear
ntg: non-transgenic
SOD1: Superoxide dismutase 1
TAR6/6: homozygous transgenic mice
TAR6: hemizygous transgenic mice
TARDBP: transactive response DNA-binding protein 43
TDP-43: transactive response DNA-binding protein 43
