# Supplementary figures and images for: mTh1 driven expression of hTDP-43 results in typical ALS/FTLD neuropathological symptoms
Source: PLoS One. 2018 May 22;13(5):e0197674. doi: 10.1371/journal.pone.0197674 (PMC5963763; doi:10.1371/journal.pone.0197674)

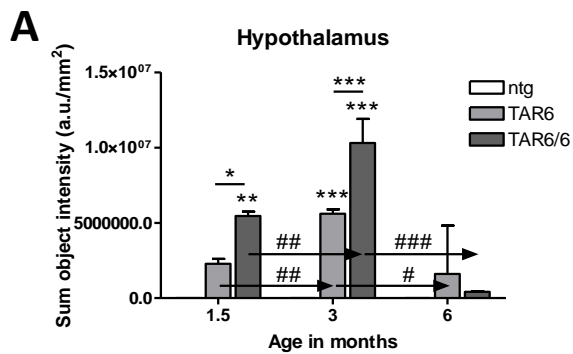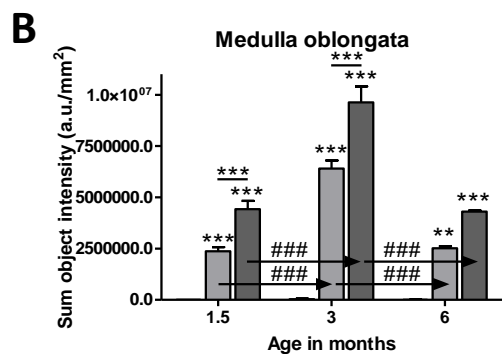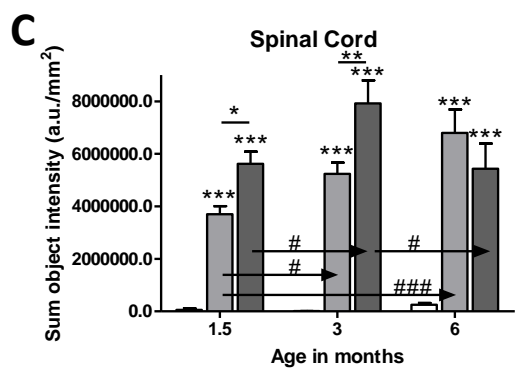

Supplement: S1 Fig — Sum object intensity of hTDP-43 expression in hypothalamus (A), medulla oblongata (B) and spinal cord (C) analyzed by immunofluorescent labeling of CNS samples. (A, B) 1.5 months: ntg: n = 4; TAR6: n = 10; TAR6/6: n = 5; 3 months: ntg: n = 5; TAR6: n = 8; TAR6/6: n = 5; 6 months: ntg: n = 3; TAR6: n = 3; TAR6/6: n = 3. (C) n as in A, B exempt: 1.5 months: ntg: n = 3; TAR6: n = 11. (A-C) Two-way ANOVA followed by Bonferroni‘s post-hoc test. Mean+SEM. *significances between genotypes, #significances between age groups. *p<0.05, **p<0.01, ***p<0.001. (PDF) [file pone.0197674.s001.pdf]

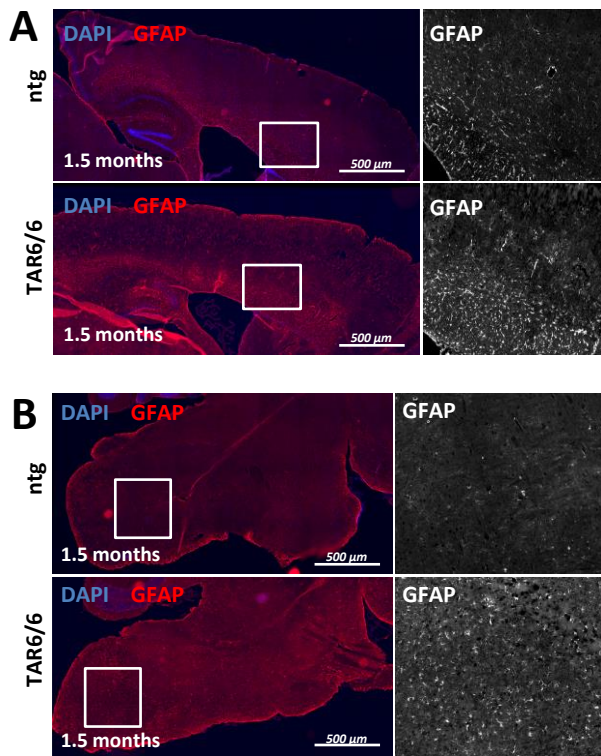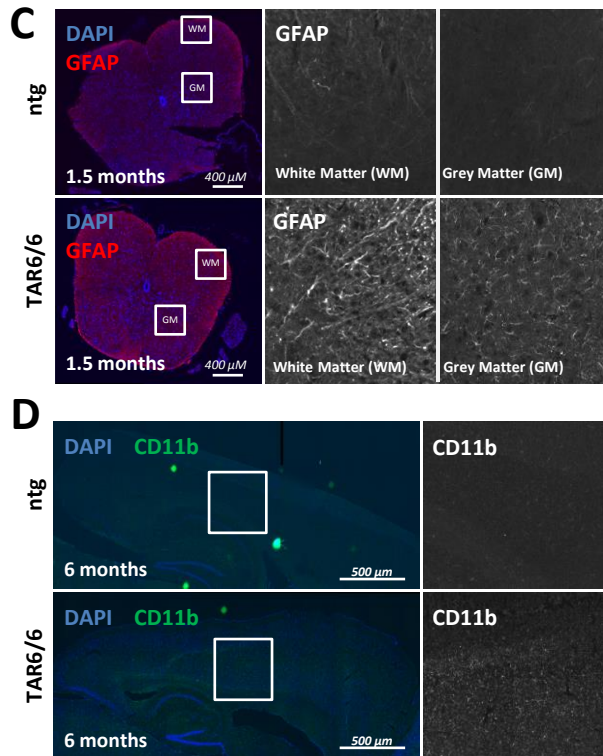

Supplement: S2 Fig — (A) Cortical, (B) medulla oblongata, (C) spinal cord GFAP expression levels labelling reactive astrocytes and (D) CD11b expression levels labeling reactive microglia. Representative images of 1.5 months old (A, B, C) and 6 months old (D) animals. Cell nuclei are stained by DAPI. (PDF) [file pone.0197674.s002.pdf]

**A**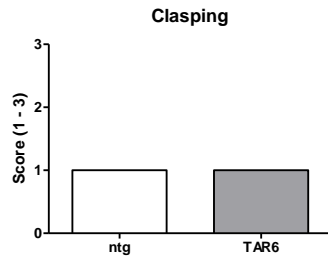**B**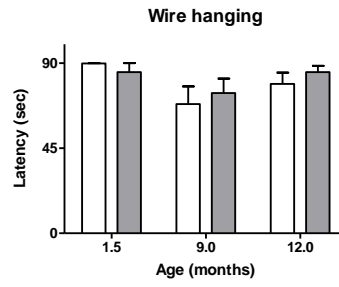**C**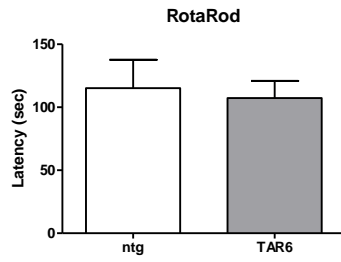**D**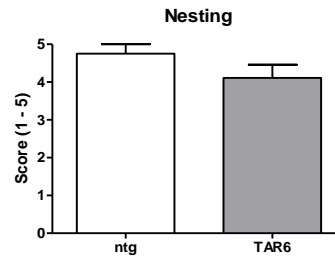**E**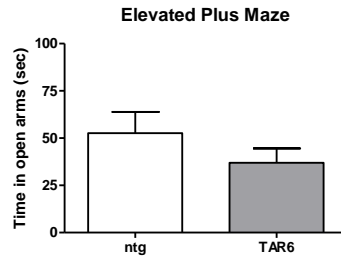**F**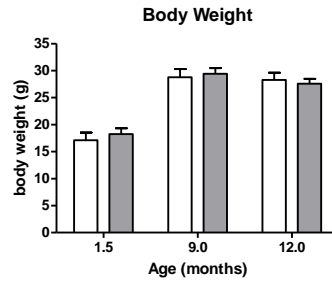

Supplement: S3 Fig — (A) Clasping behavior scored from 1–3 of 1.5 months old TAR6 mice (score of 1: normal clasping). (B) Latency to fall in the wire suspension test in seconds of 1.5, 9 and 12 months old TAR6 mice. (C) Latency to fall from the rotating rod in the Rota Rod test of 1.5 months old TAR6 mice. (D) Nesting behavior score of 1.5 months old TAR6 and TAR6/6 mice (high score: perfect nest). (E) Time in seconds 1.5 months old TAR6 mice spent in the open arms of the elevated plus maze. (F) Body weight in gram of 1.5, 9 and 12 months old TAR6 mice. (A, C) TAR6: n = 11, ntg: n = 7; (E) TAR6: n = 11, ntg: n = 6. (D) TAR6: n = 9, ntg: n = 4, TAR6/6: n = 4. (B, F) 1.5 months: TAR6: n = 11, ntg: n = 7; 9 months: TAR6: n = 15, ntg: n = 12; 12 months: TAR6: n = 15, ntg: n = 13; Mean+SEM. A: One sample t-test; C-E: unpaired t-test. B,F: Two-way ANOVA; A-F: non significant (p>0.05). (PDF) [file pone.0197674.s003.pdf]
