# Supplementary material for: mTh1 driven expression of hTDP-43 results in typical ALS/FTLD neuropathological symptoms
Source: PLoS One. 2018 May 22;13(5):e0197674. doi: 10.1371/journal.pone.0197674 (PMC5963763; doi:10.1371/journal.pone.0197674)
Supplement: S1 Table — (PDF) [file pone.0197674.s004.pdf]

Figure 1

Fig.1B: Quantification of total TDP-43 in the brain

Age	ntg			TAR6/6		
1.5	1.235423	1.098233	0.666344	1.879156	1.386271	1.429836
3	1.213704	0.715009	0.703616	2.086677	1.712153	1.7758
6	0.930674	0.703521	1.308622	2.66174	1.43329	1.97808

Fig.1C: Quantification of CTF-35 in the brain

Age	ntg			TAR6/6		
**1.5**	0	1.609298	1.390702	3.92698	5.555732	5.636176
**3**	0	3.738176	1.36563	3.727817	7.024968	3.21647
**6**	0	0.822199	1.367055	4.059849	6.138003	5.304742

Fig.1D: Quantification of human TDP-43 in the brain

Age	ntg			TAR6/6		
**1.5**	0	0	0.067398	1.308923	0.917266	0.806004
**3**	0	0.001635	0.042608	1.298348	0.934079	0.894071
**6**	0.015737	0.021057	0.057283	1.483992	0.895477	0.999733

Fig.1F: Quantification of total TDP-43 in the hippocampus

Age	ntg			TAR6/6		
**1.5**	0.436296	1.374862	1.188842	2.165639	1.751215	2.297299
**3**	1.018149	1.959313	1.30973	2.148077	2.44763	2.578893
**6**	1.099939	2.006237	1.371909	2.617591	3.179204	2.52124

Fig.1G: Quantification of total CTF-35 in the hippocampus

Age	ntg			TAR6/6		
**1.5**	0	2	0	11.77444	18.86648	22.88131
**3**	2.133286	8.110545	3.270155	12.37112	19.42252	19.60266
**6**	4.829227	11.91547	4.733284	18.62531	22.81102	17.26801

Fig.1H: Quantification of human TDP-43 in the hippocampus

Age	ntg				TAR6				TAR6/6			
**1.5**	0.08	0.03			0.94	0.83	0.73	0.92	1	1.06	1.11	
**3**	0.04	0.01	0.08		0.93	0.91	0.78		1.4	1.25	1.2	
**6**	0.05	0.03	0		0	0	0		1.55	1.1	1.07	

Fig.1J: Quantification of total TDP-43 in the spinal cord

Age	ntg			TAR6/6		
**1.5**	0.934892	0.862916	1.202193	1.716095	1.824285	1.752267
**6**	0.350827	0.519942	0.814965	1.220447	1.646701	1.266007

Fig.1K: Quantification of CTF-35 in the spinal cord

Age	ntg			TAR6/6		
**1.5**	0.484719	1.376899	1.138382	10.2822	5.093426	10.83012
**6**	2.542313	1.873982	1.768126	9.173911	1.514089*	11.13001

Fig.1L: Quantification of human TDP-43 in the spinal cord

Age	ntg		TAR6/6	
**1.5**	0.01	0.05	0.85	0.91
**6**	0.01	0.06	0.42	0.32

Figure 2

Fig. 2A: Quantification of hTDP-43 IR area in the hypothalamus

1.5 m ntg					1.5 m TAR6					1.5 m TAR6/6					3 m ntg					3 m TAR6					3 m TAR6/6					6 m ntg					6 m TAR6					6 m TAR6/6				
0*	0*	0*	0*	0*	2.24762	2.03115	0*	0*	0*	3.07638	0*	0*	0*	0*	0	0*	0*	0*		3.52347	1.78224	0*	0*	0*	5.38057	3.93476	0*	0*	0*	0.63*	0*	0*	0*	0*	0.87913	0*	0*	0*	0*	4.81068	2.4629	0*	0*	0*
0*	0*	0*	0*	0*	2.95658	2.27958	0*	0*	0*	0*	3.43002	0*	0*	0*	0.00142	0	0*	0*	0*	4.27095	3.31188	0*	0*	0*	4.26148	0*	0*	0*	0*	0.00141	0*	0*	0*	0*	1.38771	0.78427	0*	0*	0*	3.55855	0*	0*	0*	0*
0*	0.00142	0*	0.04*	0*	1.19617	0*	0*	0*	0*	4.42901	3.10649	0*	0*	0*	0.01907	0.00473	0*	0*	0*	3.05642	3.50199	0*	0*	0*	4.55247	5.98448	0*			0	0	0*	0*	0*	1.35901	0*	0*	0*	0*	1.98*	1.04*	0*	0*	0*
0.01914	0.00195	0*	0*	0*	0.69594	1.18494	0*	0*	0*	4.74665	2.02573	0*	0*	0*	1.89*	0	0*	0*	0*	3.0028	3.26148	0*	0*	0*	0*	0*	0*																	
					1.6559	0.62043	0*	0*	0*	1.91*	1.42*	0*	0*	0*	0.01742	0	0*	0*	0*	4.36816	2.92277	0*	0*	0*	4.01535	4.33529	0*	0*	0*															
					2.40768	0*	0*	0*	0*											2.8499	3.8876	0*	0*	0*																				
					1.49462	1.69012	0*	0*	0*											3.04159	2.869	0*	0*	0*																				
					2.19235	0*	0*	0*	0*											5.61211	2.76403	0*	0.01*	0*																				
					0.34323	1.71432	0*	0*	0*																																			
					1.43375	0*	0*	0*	0*																																			

Fig. 2B: Quantification of hTDP-43 IR area in the Medulla Oblongata

1.5 m ntg					1.5 m TAR6					1.5 m TAR6/6					3 m ntg					3 m TAR6					3 m TAR6/6					6 m ntg					6 m TAR6					6 m TAR6/6				
0.00075	0	0	0*	0*	0.97024	2.13231	1.44739	0*	0*	2.96035	3.55633	3.06192	0*	0*	0	0.01526	0	0		2.41832	2.38348	3.37432	0*	0*	2.64416	3.02604	3.11188	3.60009	0*	0.01317	0.02287	0,01*	0*	0*	1.39193	1.74184	0*	0*	0*	2.36415	2.31408	3.41593	0*	0*
0*	0*	0	0	0*	1.63459	2.60887	1.88093	0*	0*	0*	3.3533	1.61885	0*	0*	0	0.02023	0	0	0*	2.76403	2.69573	2.79334	4.29865	0*	3.22729	2.91736	3.00066	3.71516	0*	0	0*	0*	0*	0*	1.86844	0*	0.88939	0*	0*	2.95706	2.93318	2.13787	0*	0*
0*	0	0	0*	0*	0.85782	1.29333	1.27699	0*	0*	2.49026	2.57698	3.66425	0*	0*	0.00148	0	0	0*	0*	0*	2.43381	2.12902	2.39706	0*		4.37095	3.77107			0	0	0	0*	0*	1.11219	1.56821	0*	0*	0*	0*	1.36*	1.39*	0*	0*
0	0	0	0*	0*	1.14231	0.83508	1.58153	0*	0*	4.42157	2.54467	2.45191	0*	0*	0*	0.04952	0	0.15268	0*	2.3275	2.87309	3.49501	3.59673	0*	4.86162	4.45284	0*																	
					2.23427	1.25039	1.64503	1.8223	0*	1.62822	2.23404	2.36096	0*	0*	0.00231	0	0	0	0*	2.59917	2.75794	3.51449	3.65736	0*	3.26484	3.05647	3.43934	3.45769	0*															
					1.49174	2.04092	1.32723	1.62714	0*											1.95846	2.48223	2.90911	3.62822	0*																				
					1.12223	2.27468	1.37011	0*	0*											0*	1.6742	2.21368	4.15928	0*																				
					3.85552	0.6401	1.46939	1.40346	0*											3.65358	2.37983	2.83293	4.20106	0*																				
					1.19845	1.72126	1.72364	0*	0*																																			
					0.89*	0*	0*	0*	0*																																			

Fig. 2C: Quantification of hTDP-43 IR area in the spinal cord

1.5 m ntg			1.5 m TAR6			1.5 m TAR6/6			3 m ntg			3 m TAR6			3 m TAR6/6			6 m ntg			6 m TAR6			6 m TAR6/6		
0*				0*	0*		4.10023	3.92108	0.00115	0	0		4.02716	3.0124	5.02063		6.03148	0.25066	0.2244	0.2195	2.7365	2.27821	2.65859	2.36339	1.89044	2.37433
0*	0*			4.92*	5.23*	4.55085	4.30384	3.00463	0.0998	0.00076	0.01174	4.95126	5.20397	5.25552	4.85646	5.48402	4.96915	0.41811	0.16773	0.34486	3.19502	2.65916	2.82895	2.90061	2.36465	1.81982
0		0	3.98298	4.22565	2.06363	4.66869	2.94434	3.92138	0.01024	0	0.00643	3.03936	3.7923	3.7867	0*	0*	0*	0.22421	0	0.15482	3.2985	3.79822	3.1149	3.39369	3.03158	3.11119
0.07007	0.14536	0.07692		3.40188	2.88969		3.10789	3.60659		0.00985	0*	3.51*	4.32748	4.51367		0*	0*									
			0*	3.22081	1.93671				0.00235	0.01333	0	0*	0*	0*	3.99286	4.71704	4.43962									
			1.98886	0.92422	2.48992							3.73182	3.80263	3.7781												
			3.06432	3.30833	3.16117							4.85*	4.65397	0*												
			3.50246	2.98043	1.18349							4.31*	4.4*													
			2.93045	2.94175	1.71758																					

Figure 3

Fig.3B: Cellular localization of total TDP-43

	ntg				TAR6				TAR6/6			
**Cyt**	3.923645	25.05096	12.04496	13.37534	47.92168	36.42477		26.92542	21.75479	85.84364	63.56861	37.57645
**Nuc**	86.53465	127.9196		85.54579	228.9773	216.8489		229.5134	230.0574	164.6462	260.8906	278.5709

Fig.3C: Cellular localization of CTF-35

	ntg				TAR6				TAR6/6			
**Cyt**	0.008343	0.008343	0.008343	0.008343	177.7287		160.9928	21.27438	130.3744	289.707	162.4111	
**Nuc**	96.58567	54.87121	103.0931	145.45	431.3109	395.645	448.4972	402.5196	552.1494	178.3127	313.3591	195.3238

Fig.3D: Cellular localization of human TDP-43

	ntg				TAR6				TAR6/6			
**Cyt**	0.002693	0.002693	0.002693	0.002693	57.37139		51.969	6.867438	42.08528	93.51835	52.42683	
**Nuc**	31.17817	17.7126	33.2788	46.95173	139.2286	127.7155	144.7764	129.9346	178.2356	57.55991	101.1533	63.05116

Fig.3F: Fractionation of total TDP-43

	ntg					TAR6/6				
**RIPA**	82.73653	121.6881	155.097	25.64466	114.8337	625.6707	599.7069	587.7355	543.0641	465.0547
**UREA**	15.72951	28.70548	75.85857	42.53232	32.59354	383.3463	333.5224	431.1494	276.336	328.3344

Fig.3G: Fractionation of CTFs

	ntg			TAR6/6		
**RIPA**	87.99545	78.63189	133.3727	494.1198	494.2175	381.2932
**UREA**	321.9989	351.9696	373.2603	349.3815	406.1366	217.6203

Fig.3H: Fractionation of human TDP-43

	ntg					TAR6/6				
**RIPA**	0.434506	0.222451	2.189162			92.72234	77.2797	118.0547	102.5351	109.4082
**UREA**	1.332624	2.613273	4.32427			56.54192	55.96396	70.60203	81.2152	56.03672

Figure 5

Fig.5A Quantification of GFAP IR area in the cortex

1.5 m ntg					1.5 m TAR6					1.5 m TAR6/6					3 m ntg					3 m TAR6					3 m TAR6/6					6 m ntg					6 m TAR6					6 m TAR6/6				
69.63	97.89	103.33	118.95	67.26	100.16	138.44	146.54	141.82	114.8	142.41	162.76	111.32	110.27	109.98			106.16	78.26	96.92	138.48	107.66	79.44	63.16	63.58	112.49	133.19	90.33	116.77	112.1	39.8	78.1	156.5	109.3	91.6	19.3				23.9	74.3	95.8	44.4	45.5	64
81.6	132.03	79.36	82.81	89.45	65.24	165.55	131.32	90.89	100.97	146.84	169.6	179.55	159.7	162.78	94.5	132.1	120.59	68.53	87.4	55.73*	125.05	164.56	68.44	76.47	160.61	139.37	95.65			69.5	126				93	21.2	16.3	73.9		78.4	97.6		93.3	121.1
101.11	148.98	109.25	76.36	91.55	101.7	128.68	120.6	183.45	105.8	198.07	187.45	186.1	197.86	189.56	89.54	80.44		87.09	106.38	94.74	105.27	98.96	81	61.34	208.97	161.08	118.56	137.95	120.04	99.4	114.8	149.1		88.6				74.3	95.8	129.2				
120.71		117.41	121.02	91.29	187.71	148.42	157.84	169.98	145.56	174.43	252.13	164.53	171.32	162.79	62.56	131.97	112.27	104.53	83.35	169.54	144.71	135.52	101.8	85.68		93.62	106.44	118.9	134.19	79.9	97.4													
					108.43	131.62	153.7	161.34	109.59	117.01	191.54	195.32	133.43	140.59							106.44	110.52	101.15			110.54	156.98	142.45	120.73															
					216.61*	212.81*	291.07*	269.36*	283.96*											45.93*	111.11	114.75	115.17	80.34																				
					47.77	293.15	319.79	145.25	134.96											104.63	105.77	89.55	91.28																					
					139.83	203.14	146.23	116.75	120.88											74.05	129.58	117.77	106.04	88.33																				
					138.79	156.85	228.51	197.81	124.2											96.94	124.72	244.29	146.31	107.74																				
					117.88	178.71	156.2	156.18	98.42																																			

Fig.5B Quantification of GFAP IR area in the Medulla oblongata

1.5 m ntg					1.5 m TAR6					1.5 m TAR6/6					3 m ntg					3 m TAR6					3 m TAR6/6					6 m ntg					6 m TAR6					6 m TAR6/6				
49.716	48.3453	132.602	28.1055		107.315	151.985	51.2644	63.7293		250.654	162.797	228.102	125.93				62.8619	109.836			65.4981	112.871	100.016		83.1942	105.568	111.827	88.0772			99.9203	135.577			36.4395	28.214				120.478	87.2443	57.6029*	49.9536*	
64.6729	137.202	49.0312				118.873	71.6299	63.402		68.6164	367.976		155.523		223.256	83.3759	14.5309*	146.734			31.5545*	69.0304	79.4119		107.989	152.557	181.268	138.79		101.385	62.051	85.2313	94.7393		48.6246	46.5936	22.7687*	67.6531		119.988	97.6238	132.355	118.525	
67.9555	110.946	176.971	97.157		78.5111	139.159	79.7702	148.325		132.632	205.325	380.934			102.965	84.1788	123.684	76.0158		44.8902	92.6533	95.7447	71.11		254.463	97.5265	176.439	206.907		96.4327	109.356	104.288	111.02		95.5392		108.659			106.57	118.279	111.292	133.079	
126.535	147.85	206.295	56.6159		92.7994	397.647	119.488	212.784		397.433	57.1767	66.6202					59.5308	104.015			69.0382	93.2407	107.766																					
					113.816		100.544	269.397		106.534	93.5186	604.239			211.425*	78.9577	108.462	138.958		90.211	73.1143	83.1595	55.1647		155.998	64.8029	77.5087	118.515																
					150.912	231.211	268.574	56.0224												106.653	37.1857*	60.4834	110.001		104.614		132.189	86.5872																
					102.519	206.479	94.8503	65.5587												80.2752	109.048	101.97	67.7891		79.9117	70.9524	185.533																	
						155.936	55.4904	92.9752												94.09	75.9491		98.1091		108.727	88.8892	130.605	80.2694																
					53.8955	94.1395	73.5807																																					
					123.535	64.3425	113.878	73.009																																				

Fig.5C Quantification of GFAP IR area in the Spinal cord

1.5 m ntg			1.5 m TAR6			1.5 m TAR6/6			3 m ntg			3 mo TAR6			3 mo TAR6/6			6 m ntg			6 m TAR6			6 m TAR6/6		
	133.818	91.4577	189.266	230.446						89.7988	142.334		159.422		115.039		206.954	96.6565			84.8063	63.5294	74.4861	94.8814	176.1	
	86.0183						400.512		129.456			195.212		214.865	124.798			113.724			123.947	84.44	82.6472	147.092	109.796	98.2293
	88.706		91.1348	142.848	104.561	614.197			101.633		140.784	230.628	143.469	146.805				89.6208			382.971*	195.684	425.291*	137.21	113.405	110.461
			125.225	183.301	98.8314	727.035	175.809	614.673	87.1809				305.643	158.446	183.587											
			297.61	347.26	575.543*	346.972		88.5857*	51.6412	57.1737																
			501.113		428.276							86.9732		271.142												
			258.422	609.843	404.15																					
				43.9375	82.2509	229.719																				

Fig.5D Quantification of CD11b IR area in the cortex

1.5 m ntg					1.5 m TAR6					1.5 m TAR6/6					3 m ntg					3 m TAR6					3 m TAR6/6					6 m ntg					6 m TAR6					6 m TAR6/6				
111.133	209.816	207.222	192.726	112.083	339.284	13.8435	278.324	285.769	170.025	58.5624	99.0041	39.7317	132.298	102.224	126.03	25.9426	122.016	107.797	105.885	175.454	24.6105	118.137	289.586	320.867	270.581	10.7782*	48.4628*	255.98	95.4501						42.7707	66.5228	206.972	66.8973	74.1014	556.19	361.464	405.063	879.425	612.479
154.901	18.5236	162.288	138.171		72.5992		73.125	93.8108	100.378	359.213	37.7204	359.584	179.604	132.082		10.5261	13.2456	69.5531	80.2369	223.015	202.86	207.618	307.276	278.158	167.946	360.071	305.762	140.752	172.525	145.294	18.0381	70.9208	93.218	15.6131	141.108	71.5623	24.9657	63.8839		1036.3	378.956		1796.74*	674.121
	94.5366	38.0225	40.1496	100.35	275.12	131.838	15.391	228.922	133.878	272.407	20.7723	148.886	155.619	29.2932		42.2173	169.199	119.524	127.665	90.3678	24.4402	243.432	320.689	263.936	204.135	95.4574	312.168		131.145	125.579	148.452	122.847	106.209		117.851		120.128	85.1658		85.5097	92.2957	105.474	74.101	133.784
100.03	17.8697	23.4014	16.9614	61.8154		33.0235	167.223	79.4563	264.457	373.56	33.3745	179.554	188.994	237.41	78.7951	30.6088		32.1055	72.3895	230.513	31.9583	231.216	243.232	234.411	199.097	351.973	261.591		42.0454*	238.748	133.985	80.9379	39.7984	60.3604										
					75.6768	47.3432	77.8958	153.469	49.8981	136.505	24.4364	221.368	107.732	111.709	176.78	18.5445	226.473	229.79	214.675		16.8617	105.085	95.9625	133.28	192.947	152.097	218.055																	
					220.534	46.7369	211.27	330.758	226.61											36.3514	171.051	223.5	279.536	105.414																				
					10.6834	397.23	334.606	284.484	317.879											122.722	194.419	152.187	75.335	53.2324																				
					14.2779	50.781	19.4358	3.74249												171.718	220.885	139.694																						
					223.082	114.125	168.962	452.532	240.036																																			
					223.64	75.911	255.039	140.696	143.298																																			

Fig.5E Quantification of CD11b IR area in the Medulla oblongata

1.5 m ntg					1.5 m TAR6					1.5 m TAR6/6					3 m ntg					3 m TAR6					3 m TAR6/6					6 m ntg					6 m TAR6					6 m TAR6/6				
140.093	67.7585	38.7061			52.9149	229.023	82.5416	52.273			74.8317	11.3791	24.2369		85.9973	191.306	182.819	356.008		98.1778	176.15	173.051	329.728		11.0723	95.228	651.907	67.7258		1366.8*	530.562*	841.881*	173.324*		71.7716	188.334	91.0441	64.6663		193.936	357.752	622.795	260.118	
0.84813	342.828	88.6888				74.3345		15.5856		36.2407	66.1372	117.51	58.6393		36.5377	40.1133	230.098	142.814	61.4288	483.713	281.633	373.993	534.812		818.667		0*	0*	0*	127.831		103.112	43.2066		132.435				0	361.81	348.526	844.597	199.835	
42.8325	83.6488	267.767	23.5788		47.5297		121.992	7.7156		45.4537	229.186	169.799	10.4998		58.4294	326.446	280.465	156.085	65.4713	114.544	408.877	148.399	122.157		92.3275	429.219	399.323	313.153	36.5287	90.4945	119.278	84.9174	36.7952		48.4467		46.8116			132.739	81.5915	117.388	61.9532	
74.7756	120.189		8.28617		7.90941	4.38498	35.4285	57.1935		88.3674	547.936	300.77	49.1478		128.337	302.637	200.369	283.742		10.7313	76.9193	304.329	448.975	70.6095	89.671	345.338	495.609	330.985	86.1838	107.281	88.0298	77.5668	35.9957	31.5467										
							38.9837	10.843		12.2679	152.857	139.198	48.9054		39.6946	281.14	379.316	639.123		70.9706	242.38	130.219		46.8434	97.2262	141.02	257.273	290.275	176.915															
					57.9738	254.594	188.151	40.1215												102.58	277.997	336.894	76.6667	209.438																				
					201.839	337.128	168.095	158.048	1.33613											344.584	196.697	44.6562	0*	70.169																				
					113.858	90.7423	19.5984	20.6057	159.603											205.483	442.879	157.378	69.6607																					
					129.645	115.561	129.609	12.0057																																				
					50.0913	253.086	167.087	9.77859																																				

Figure 6

Fig.6A: Quantification of ChAT positive neurons in the cervical spinal cord ventral horn

ntg				TAR6/6			
9	7.3	8	9.25	9.25	10.75	8	4
13	*19.75**	9	*14**	11	8	4	6
8	7	12	11.5	5.6	8.4		
9.5	8	10	11.3				

Fig.6A: Quantification of ChAT positive neurons in the lumbar spinal cord ventral horn

ntg				TAR6/6			
12	14.7	8.25	9.25	5.5	6.5	8.5	9.5
		15	11.3	8.4	7.8	4.6	6.3
		12	12	14.3	12.3	6	5.3

Figure 7

Fig.7A: Clasping score

Age	ntg																TAR6/6															
1.5	1	1	1	1	1	1	1										1	2	2	2	2											
3	1	1	1	1	1	1	1	1	1	1	1	1	1	1	1	1	2	1	2	2	1	1	2	1	2	2	2	2	3	1*	1*	1*

Fig.7B: Wire hanging time

Age	nTg																TAR6/6															
1.5	90	90	90	90	90		89	90									9	36		5	12	33										
3	90	90	90	83	90	90	90	90	90	90	90	90	90	90	81	90	27	31	13	5	12	90*	90*	80	86	90*	10	36	51	40	7	90

Fig.7C: RotaRod latency to fall

Age	ntg																TAR6/6															
1.5	104	180	180	117	21	59	145										31	110	83	106	51											
3.5	154	106	183	87	18	180	107										51	104	12	90	9	130*	27	3	113	23*						
3.75	180	108	176	161	87	106	180	147	94	183	76	98	164	115	170	124	70	15	71	13	55	22	132	29	131*	15	17	27	22	161*	176*	
4	176	180	140	176	150	66	125	90	99	106	180	145	125	170	174	181	39	25	98	67	52	3	143*	48	41	59	75	170*	175*			
4.25	178	168	149	187	127	177	183	99	120	119	124	103	177	180	174	146	100	58	103	53	30	145*	42	23	43	53	172*	152*				

Fig.7D: Nesting score

ntg	TAR6/6
2	1
4	1
5	1
3	1
1	1
4	2
4	1
4	1
4	2
4	2
4	2
4	4*
3	2
3	3*
2	4*
4	

Fig.7E: Elevated Plus Maze time in open arms

Age	ntg																TAR6/6															
1.5	62.92	70.44	9.32	44.16	88.44	40.44											158.2	153.36	143.72	174.08	249.76											
4.5	31.48	4.96	4.2	0	0.56	34.72	40.52	0.76	0	0	22.12	6.24	0	0.8	140.64	52.2	239	112.72	132.48	79.52	8.52*	124.2	234.6	83.96	138.8	1.28*	96*					

Fig.7F: Body Weight

Age	ntg																		TAR6/6																	
1.5	15.6	15.1	13.9	17.3	13.4		21.5	23													14.9	13		20.6	20.6	19.4										
3.25							20.9	20.5	25.1	26.8	27.4	26	26.4	20.3	22	20.8	22	20.8							24.4	11.2	17.1	20.1	23	15.8		11				
3.5	17.2	25.8	21.9	23.1	22.7	23.6	21.3	21.3	25.5	27.3	27.1	26.4	26.6	20.7	22.9	20.7	22.9	20.7	10.8	20.9	17.7	17.7	19.7	20.2	21	9.4	16.6	19.8	23.6	16.6						
3.75	17.5	25.1	20.2	22.1	19.1	23.5	21.6	21.5	25.5	27.4	27.6	26.8	27.5	21.7	22	20.7	22	20.7	10.8		18	16.2	17.3	20.4	23.9		17.8	19.8	24.3	16.7						
4	18.5	24.9	19.4	20.9	21.6	24.8	22.1	21.5	26.4	27.8	27.6	26.6	27	21.7	22.4	21.8	22.4	21.8	10.2		19.1			21	25.7		19.6	20	23.7	15.2						
4.25	18.1	24.8	20.5	20.7	22.2	24.6	21.6	21	25.5	27.5	26.9	26.6	26.4	21.8	22.7	21.7	22.7	21.7	9.8		19.6			20.1	26.3		19.7	20.5	24.2	18.2						

S1 Figure

S1A Figure: Quantification of human TDP-43 sum object intensity in the hypothalamus

Age	1.5			3			6		
	Mean	SD	N	Mean	SD	N	Mean	SD	N
**ntg**	6513	7081	2	4749	6271	5	785.3	1111	2
**TAR6**	2280000	1030000	10	5600000	834470	8	1610000	5550000	3
**TAR6/6**	5450000	579866	4	10300000	3200000	4	423247	48346	2

S1B Figure: Quantification of human TDP-43 sum object intensity in the Medulla oblongata

Age	1.5			3			6		
	Mean	SD	N	Mean	SD	N	Mean	SD	N
**ntg**	71.89	143.8	4	33344	68785	5	6631	11485	3
**TAR6**	2370000	593653	9	6400000	1130000	8	2520000	156443	3
**TAR6/6**	4420000	901649	5	9630000	1740000	5	4300000	81233	2

S1C Figure: Quantification of human TDP-43 sum object intensity in the Spinal cord

Age	1.5			3			6		
	Mean	SD	N	Mean	SD	N	Mean	SD	N
**ntg**	58976	83405	2	7785	9873	5	251533	122816	3
**TAR6**	3700000	821317	7	5240000	1050000	6	6800000	1550000	3
**TAR6/6**	5620000	930462	4	7920000	1530000	3	5430000	1670000	3

S1 Figure

S3A Figure: Clasping Score

ntg	TAR6
1	1
1	1
1	1
1	1
1	1
1	1
1	1
	1
	1
	1
	1

S3B Figure: Wire hanging time

Age	nTg														
**1.5**	90	90	90	90	90		89	90							
**9**	90	90	90	90	90	90	90	20	21	36	24	90			
**12**	90	90	90	90	90	20	90	90	90	48	81	68	90		

Age	TAR6														
**1.5**	90	90	90	90	90	90	90	90	90	38	90				
**9**	90	90	90	90	90	90	90	90	37	8	14	64	90	90	90
**12**	90	90	90	90	90	90	90	90	90	90	90	44	65	90	90

S3C Figure: Rotarod time to fall

ntg	TAR6
104	53
180	179
180	180
117	97
21	57
59	58
145	125
	86
	94
	119
	133

S3D Figure: Nesting Score

ntg	TAR6
4	3
5	2
5	5
5	5
	4
	5
	5
	4
	4

S3E Figure: Elevated Plus Maze time in the open arms

ntg	TAR6
88.44	38.56
40.44	52.04
62.92	102.36
70.44	28.56
9.32	31.04
44.16	35.4
	22.32
	18.48
	23.2
	6.52
	47.8

S3F Figure: Body Weight

Age	ntg														
**1.5**	15.6	15.1	13.9	17.3	13.4		21.5	23							
**9**	25.1	26.5	26.7	21.3	26.2	25.3	31.1	37.7	34.2	23.1	33.7	34.4			
**12**	20.3	25.2	22.1	25.4	22.4	33.5	28.8	28	32.3	33	33.2	34.6	28.8		

Age	TAR6														
**1.5**	14.3	12.7	14.8	15.9	16.1		19.6	20.3	20.3	23.2	22.3	21.3			
**9**	26.5	26.1	25.3	25.2	23.6	26	25.8	30.7	33.5	31.4	35.2	37	31.2	31.8	31.8
**12**	25.4	24.3	26.5	26.4	24.2	25.2	27.7	25.6	32.2	30.5	29.5	33.7	33.3	22.8	26.5
